# Label-Free Proteomics Reveals Decreased Expression of CD18 and AKNA in Peripheral CD4^+^ T Cells from Patients with Vogt-Koyanagi-Harada Syndrome

**DOI:** 10.1371/journal.pone.0014616

**Published:** 2011-01-28

**Authors:** Liming Mao, Peizeng Yang, Shengping Hou, Fuzhen Li, Aize Kijlstra

**Affiliations:** 1 Laboratory of Ophthalmology, Chongqing Eye Institute, The First Affiliated Hospital of Chongqing Medical University, Chongqing, People's Republic of China; 2 The Department of Ophthalmology, University of Maastricht, Maastricht, The Netherlands; University of Miami, United States of America

## Abstract

Vogt-Koyanagi-Harada (VKH) syndrome is a systemic autoimmune disease. CD4^+^ T cells have been shown to be involved in autoimmune diseases including VKH syndrome. To screen aberrantly expressed membrane proteins in CD4^+^ T cell from patients with active VKH syndrome, blood samples were taken from five patients with active VKH syndrome and five healthy individuals. A label-free quantitative proteomic strategy was used to identify the differently expressed proteins between the two groups. The results revealed that the expression of 102 peptides was significantly altered (p<0.05) between two groups and matched amino acid sequences of proteins deposited in the international protein index (ipi.HUMAN.v3.36.fasta). The identified peptides corresponded to 64 proteins, in which 30 showed more than a 1.5-fold difference between the two groups. The decreased expression of CD18 and AKNA transcription factor (AKNA), both being three-fold lower than controls in expression identified by the label-free method, was further confirmed in an additional group of five active VKH patients and six normal individuals using the Western blot technique. A significantly decreased expression of CD18 and AKNA suggests a role for both proteins in the pathogenesis of this syndrome.

## Introduction

Vogt-Koyanagi-Harada (VKH) syndrome is an autoimmune disorder mainly affecting systemic melanocytes including those in the eyes, meninges, ears, skin, and hair [1]. It is one of the most common uveitis entities in China as well as in the Far East of Asia. The uveitis seen in this syndrome is mostly characterized by a chronic granulomatous inflammation with recurrent episodes [2]. Although the mechanisms involved in VKH syndrome are not fully elucidated, previous reports have showed that CD4^+^ T cells sensitive to melanocytes are responsible for the development of VKH syndrome [3], [4]. Recent studies have shown that molecules related to CD4^+^ T cell function, including Fas/FasL [5], [6], T-bet [7], IFN-γ, RORγt and IL-17 [7], [8], are involved in the pathogenesis of this syndrome. As CD4+ T cells exert their role principally through a variety of receptors, adhesion molecules,transport proteins and costimulatory molecules,which have been found mainly in the plasma membrane or endomembrane systems, our study focused on the differentially expressed proteins in the CD4+ T cell membrane of VKH patients.

Proteomics provides important tools for identifying molecules involved in both normal and pathological processes [9], [10], [11], [12], [13], [14], [15]. A recently established label-free strategy [16], [17] has been shown to offer a more robust protein identification and quantitation, easy automation and large-scale analysis with greater efficiency as compared to conventional two dimensional electrophoresis (2-DE) approaches [18], [19], [20], [21]. In the present study, we investigated the differentially expressed membrane proteins in active VKH patients using this label-free proteomic method. Western blot technique was used to validate the proteomic results. Our results showed a significantly decreased expression of CD18 and AKNA in CD4^+^ T cells from patients with active VKH syndrome.

## Materials and Methods

### Clinical Samples

Ten active VKH patients and eleven healthy individuals were included in the present study. CD4^+^ T cells from five active VKH patients and five healthy individuals were used for label-free proteomics analysis. CD4^+^ T cells from another group of five active VKH patients and six controls were used for a validation study.The diagnosis of VKH syndrome was made according to the criteria revised for VKH syndrome by an international nomenclature committee [2]. The patients included in our study had not been systemically treated with any immunosuppressive agent for at least one week prior to blood sampling. The mainly clinical features of VKH patients were shown in Table 1. The healthy individuals are sex and age matched with VKH patients showing not any past history of infectious or chronic diseases. An informed consent (written) was obtained from all patients and the procedures had been approved by the Ethics Committee of the First Affiliated Hospital of Chongqing Medical University.

**Table 1:** The clinical features of the investigated patients with VKH disease.

Patient number	Sex	Age	Ocular Manifestation						Visual Acuity		Extraocular Findings			
			Mutton-KPs	Aqueous cells	Iris nodules	posterior synechia	Sunset glow fundus	Multiple peripheral chorioretinal lesions	od	os	Meningismus	Tinitus	Alopecia and poliosis	Vitiligo
Screening														
1	Male	33	++	+++			present	present	0.25	0.1	+	-	+	+
2	Male	34	++++	++		present	present		0.5	0.5	+	+	+	-
3	Male	39	++	++		present	present	present	0.08	0.06	-	+	+	+
4	Male	24	+++	++	present	present	present	present	0.06	0.05	+	+	+	-
5	Female	34	++	++	present	present	present		0.8	1.0	-	+	+	+
Validation														
6	Male	18	++	++		present	present		0.7	0.6	-	+	+	-
7	Female	27	++	+++		present	present	present	0.6	0.01	+	-	+	-
8	Female	50	++	++	present		present	present	0.3	0.05	+	+	+	-
9	Male	46	++	+++		present	present		1.0	0.8	-	+	+	+
10	Male	41	+++	++	present	present	present	present	0.2	0.5	+	+	+	+

### CD4^+^ T Cell isolation and flow cytometry

The PBMCs were prepared from heparinized blood by Ficoll-Hypaque density-gradient centrifugation and were rinsed for three times in phosphate-buffered saline (PBS). Peripheral CD4^+^ T cells were purified using human CD4 microbeads according to the manufacturer's instructions (Miltenyi Biotec, Palo Alto, Calif). Briefly, PBMCs were suspended in 80 µl of PBS containing 0.5% bovine serum albumin (BSA) and 2 mM ethylenediamine tetraacetic acid (EDTA) per 10^7^ total cells. A volume of 20 µl of CD4 microbeads was added to this suspension and incubation was performed for 15 min at 4°C. The cells were then washed in 2 ml of PBS containing 0.5% BSA and 2 mM EDTA and applied to a magnetic column on a MiniMACS separation unit (Miltenyi Biotec, Palo Alto, Calif). The CD4^+^ T cell fraction was collected and used in subsequent experiments. For flow cytometric analysis, aliquots of 1×10^6^ PBMCs and isolated CD4^+^ T cells were stained with PE-cy7-conjugated monoclonal antibody (mAb) against human CD4 and appropriate isotype controls (eBioscience, San Diego, CA, USA) for 30 min at 4°C in the dark. Flow cytometric analysis was performed using FACS Calibur and CellQuest software (BD Biosciences, SanJose, CA).

### Membrane Protein Preparation

Membrane proteins were extracted using a membrane protein extraction kit (Merck KGaA, Darmstadt, Germany) according to the manufacturer's instructions. Briefly, 5×10^6^ CD4^+^ T cells were pelleted by centrifugation at 300 g for five min. The supernatant was carefully removed and discarded. Reagent A (150 µl) was added to the cell pellet and a homogeneous cell suspension was prepared by pipetting up and down and was then incubated for 10 min at room temperature with occasional vortexing. The lysed cells were placed on ice. One part of reagent B with two parts of reagent C were mixed. A total amount of 450 µl of the mixed reagent B and C was added to each tube of lysed cells and then vortexed. The tubes were incubated on ice for 30 min with occasional vortexing. After centrifuging at 10,000 g for 3 min at 4°C, the supernatant was transferred to a new tube and was incubated for 10 min at 37°C to separate the membrane protein fraction. The tubes were then centrifuged at room temperature for 2 min at 10,000 g to isolate the hydrophobic fraction from the hydrophilic fraction. The hydrophilic phase (top layer) was removed carefully from the membrane-enriched phase (bottom layer). The protein concentration of the hydrophobic protein was determined using the protein assay kit (Bio-Rad, Hercules, CA). The protein was aliquoted and stored at −80°C until use.

### Protein Digestion

Protein digestion was performed as follows. The protein concentration was adjusted to 5 ug/ul with lysis buffer. The aforementioned membrane protein was chemically reduced for 2.5 h at room temperature by adding DTT to 10 mM, and then carboxyamidomethylated in 50 mM iodoacetamide for 40 min at room temperature in the dark. Endoprotease LysC (Roche, Indianapolis, IN) was added to a final substrate: enzyme ratio of 100∶1 (w/w), and the reaction was incubated at 37° for 3 h. The urea concentration in protein samples was adjusted to 1.5 M with 25 mM NH_4_HCO_3_, and then 2 µg of trypsin was added to a final substrate: enzyme ratio of 50∶1 (w/w). The trypsin digest was incubated at 37° for 20 h. The peptide mixture was acidified by formic acid to 0.1% for further MS analysis.

### Analysis by HPLC and MS

Separation of the trypsin-digested peptides was performed with the Ettan MDLC system (GE Healthcare, Piscataway, NJ). Peptide samples were first desalted through a Zorbax 300SB-C_18_ peptide traps (Agilent Technologies, Wilmington, DE) and then separated by a 0.15 mm*150 mm (RP-C_18_) column (Column Technology Inc, Fremont, CA) with micro spray mode. An aqueous 0.1% formic acid solution was used as phase A and a solution of 0.1% formic acid and 84% acetonitrile was used as phase B. The peptides were separated with a linear gradient of 4%∼50% mobile phase B over 2 hours at a flow rate of 1300 nl/min. The column temperature was maintained at 170 °C.Both pooled samples were analyzed in triplicate.

The separated peptides were analyzed on a Finnigan LTQ linear ion trap mass spectrometer (Thermo Finnigan, San Jose, CA) equipped with a nanoelectrospray ion source [16], [17]. The injection voltage was set to 3.0 kV and the normalized collision energy was set to 35.0. The number of ions stored in the ion trap was regulated by the automatic gain control. Each scan cycle consisted of one full scan mass spectrum (m/z 0∼1600) collected in profile mode. Five MS/MS events were recorded in centroid mode with the dynamic exclusion settings: repeat count 2, repeat duration 30 s, exclusion duration 90 s. MS/MS spectra were searched against the IPI human protein database (version 3.36; 69012 entries) and analyzed using SEQUEST of BioworksBrowser rev. 3.1. The search was performed with one missed cleavage permitted. The fixed and variable modifications were set to Carbamidomethyl (C) and oxidation (M) respectively. The mass tolerance for precursor ions was set to 3.0 Da. The mass tolerance for fragment ions was set to 1.0 Da. The peptide matches were filtered based on cross-correlation scores (Xcorr) of 1.9, 2.2 and 3.75 for charge states 1+, 2+, and 3+, respectively. All the peptides with different charges were translated to single charge and expressed as “MH+” (Supplemental Table 1). To determine the false discovery rate (FDR), the data set was searched against a sequence-reversed decoy IPI human version 3.36 database using the same search parameters. FDR was calculated using the formula reported by Xue et al [17]: FDR =  Number of false peptides/(Number of true peptides+Number of false peptides)×100%.

### Label-free Detection and Quantitation of Peptides

The peaklist of peptides was generated using DeCyder MS software version 1.0 (GE Healthcare, Piscataway, NJ) and the quantitative analysis of peptides was performed. Peptide detection, elution profile comparison, background subtraction and peptide quantitation were carried out on the full scan precursor mass spectra in fully automatic mode. Peptide quantitation is based on MS signal intensities of individual LC-MS analyses. Different signal intensity maps were matched using the pepmatch module and then the peptide quantitative results were acquired. As there was no internal standard, the intensity distributions for all peptides detected in both samples were used for normalization. Throughout these studies the mass tolerance in the software was set to 0.5 atomic mass unit and the retention time tolerance was set to 2 min.

### Western blot analysis

Total protein of CD4^+^ T cells from five active VKH patients and six healthy individuals were prepared for Western blot analysis. Ten micrograms of protein from each specimen were used for SDS-PAGE. The gels were then transferred to a PVDF membrane. Membranes were incubated with antibodies at dilutions of 1∶1000 for both anti-human CD18 mAb (R&D systems, Minneapolis, MN) and anti-human AKNA mAb (Genway Biotech, San Diego, CA). Proteins were detected using the Phototope-HRP western blot detection system (Cell Signaling, Danvers, MA).

### Statistical analysis

For differential analysis of peptides from both VKH patients and normal controls, student's *t*-test was performed using the Decyder MS software. A p-value of less than 0.05 was considered as a significant difference. In the validation study, the immunoblot bands were quantified using Bio-rad quantity one 1-D analysis software. Band density was normalized to that of an internal control, β-actin. The differences of immunoblot band intensities between both groups were analyzed by student's *t*-test using SPSS software (version 13.0). A *p*-value of less than 0.05 was considered as statistically significant.

## Results

### CD4^+^ T cell isolation

**Figure 1:**
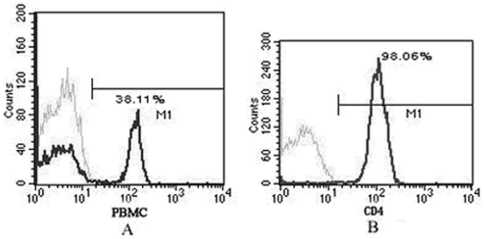
Representative flow cytometric map of CD4^+^ T cells isolated from peripheral blood of active VKH patients. The PBMC and freshly isolated CD4^+^ T cells were stained with the indicated markers using fluorescence-labeled mAb and analyzed by flow cytometry. Before sorting, the ratio of CD4^+^ T cells in PBMC was 38.11% (A). After microbeads based sorting, the purity of CD4^+^ T cells was as high as 98.06% (B).

CD4^+^ T cells were sorted from peripheral blood of ten active VKH patients and eleven healthy individuals using CD4 microbeads. The purity of CD4^+^ T cells identified in both patients and controls was 98% (Figure 1). The obtained CD4^+^ T cells from both groups were subjected to label-free quantitative proteomic analysis followed by Western blot validation.

### Data quality evaluation

Before conducting the relative protein profiling analysis between the two pooled samples from VKH patients and normal controls, several quality control measures were performed on the replicates of both pools to determine analytical reproducibility. The mass precision of the extracted peptide components was within 5 parts per million (ppm) of mass error. The quality control analysis revealed similar results between patients and controls concerning the median and average mass errors (1.90 vs 2.80 ppm), intensity errors (2.10% vs 1.95%), and retention time (0.70% vs 0.50%).

Reproducibility between duplicate runs was evaluated using a binary comparison map. The horizontal (x) axis represents the distribution of peak intensities of the first run, and the vertical (y) axis represents that of the second run. The average intensity correlation coefficient (CC) between the two runs was 0.92. A representative binary comparison map is shown in Figure S1. The expected distribution of the duplicate runs showed no obvious change. These quality control measurements were performed on all runs and both experimental pools.

### Relative quantitation of peptides in total membrane protein extracts

**Figure 2:**
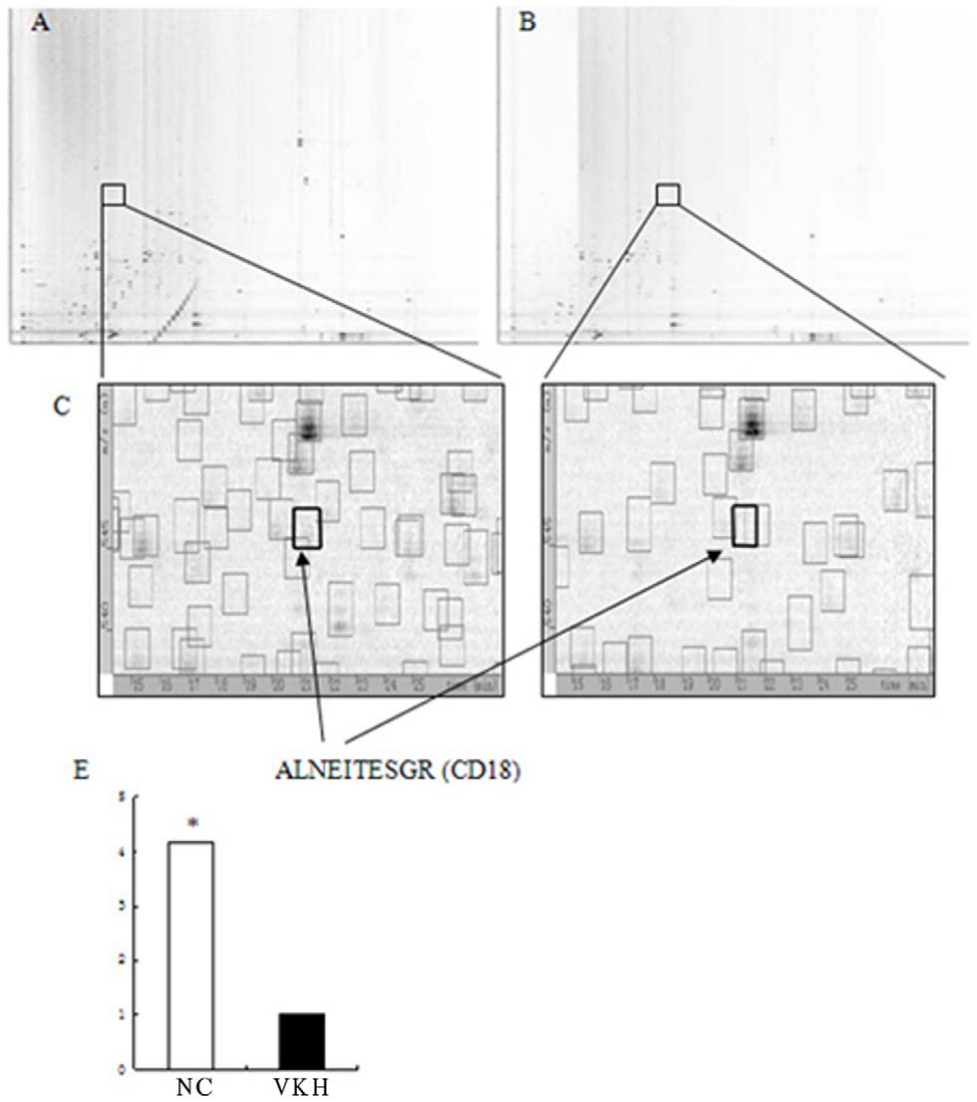
Decyder MS intensity graphs of CD18 derived from LC-MS/MS analysis of a total membrane protein extracted from both normal group (A) and VKH group (B). The location of peptide ALNEITESGR in both samples was labeled with square frames in the total graphs and the magnified graphs (C and D). Statistical analysis showed that the different expression of peptide ALNEITESGR was significant between VKH group and normal group (E).

The digested membrane proteins of CD4^+^ T cells prepared from active VKH patients and normal controls were analyzed by LC-MS/MS. Base peak ion chromatograms were acquired in profile mode to get enough data points for evaluation by DeCyder MS. A representative base peak ion chromatogram from the analysis of the VKH pool is shown in Figure S2. The other runs displayed similar profiles. The MS files were converted to signal intensity maps, 2-D representations with m/z on the y axis and retention time on the x axis. A representative intensity map (CD18) is shown in Figure 2, A and B. The density of the grayscale pattern was proportional to the signal intensity of a peptide peak at a certain m/z, retention time, and charge state. The signal intensity maps were analyzed using the PepDetect module of DeCyder MS to detect and quantitate the peptide ions. Since each sample was analyzed in triplicate, peptides from six signal intensity maps were matched and analyzed. As the peptide content reflects the protein level, relative quantitation for corresponding proteins was obtained after importing identification results. A *p*-value of 0.05 was used as a threshold to identify significantly up- or down-regulated expression. The MS/MS data was collected in centroid mode and was transferred to TurboSEQUEST software for protein identification.

In total 354 peptides were identified to be significantly different between both groups, in which 102 could be sequenced and which corresponded to 64 proteins. A FDR of 3.77% at the peptide level was obtained by searching against the sequence-reversed decoy IPI human database, suggesting a high fidelity of this strategy. Thirty proteins showed a difference of at least 1.5-fold, when comparing active VKH patients with normal controls. Of these, eight proteins were identified based on two or more unique peptides (Table 2) and in 22 the identification was based on a single peptide (Table 3). All the identified peptides and their sequences of each protein in both samples are listed in the Table S1. For single-peptide-based identifications, the MS/MS spectra appropriately labeled were listed in Figure S3.

**Table 2:** The differently expressed proteins identified based on two or more peptides in CD4^+^ T cells between active VKH patients and normal individuals.

Gene symbol	Swiss-prot accession No.	Identified proteins	Expression Ratio^a^ (VKH:NC)	Xcorr^b^	t-test p	MH+ (Da)	Mass stdev (Da)	Identified Peptide
**Up-regulated in VKH**								
VIM	B0YJC4	Vimentin	2.8	2.42	0.00004	1060.7690	0.08313	159 R.QVDQLTNDK.A 169
				5.53	0.00002	1169.9804	0.11453	129 K.ILLAELEQLK.G 140
				2.27	0.00061	1428.8390	0.13478	50 R.SLYASSPGGVYATR.S 65
				3.16	0.00031	1115.8539	0.14490	105 K.VELQELNDR.F 115
				3.36	0.00032	1323.8875	0.13155	196 R.EEAENTLQSFR.Q 208
				3.99	0.00045	1406.1375	0.30727	223 K.VESLQEEIAFLK.K 236
				3.30	0.00030	1533.9759	0.26803	222 R.KVESLQEEIAFLK.K 236
				3.30	0.00029	1312.8985	0.35813	207 R.QDVDNASLAR.L 218
				2.26	0.00061	1226.9591	0.20369	294 K.FADLSEAANR.N 305
KRT10	P13645	Keratin, type I cytoskeletal 10	2.0	2.26	0.00449	993.8803	0.14940	237 K.YENEVALR.Q 246
				2.49	0.00409	1435.8816	1.01701	439 K.IRLENEIQTYR.S 451
				3.40	0.00108	2083.0051	0.17698	422 R.AETECQNTEYQQLLDIK.I 440
				2.27	0.02983	1262.8610	0.14280	450 R.SLLEGEGSSGGGGR.G 465
				3.03	0.00403	1031.5372	0.57250	257 R.VLDELTLTK.A 267
				3.84	0.00012	1494.0186	0.19081	322 R.SQYEQLAEQNRK.D 335
				2.26	0.00448	1236.916	0.16415	165 R.ALEESNYELEGK.I 178
ATP5B	P06576	ATP synthase subunit beta, mitochondrial precursor	1.8	2.26	0.00433	1988.1477	0.17359	387 R.AIAELGIYPAVDPLDSTSR.I 407
				3.57	0.00622	975.7282	0.15882	201 K.IGLFGGAGVGK.T 213
				2.67	0.00022	1088.6165	0.50286	188 K.VVDLLAPYAK.G 199
ACTA2	P62736	Actin, aortic smooth muscle	1.8	3.83	0.01604	1354.8744	0.12513	52 K.DSYVGDEAQSKR.G 65
				3.12	0.00290	1198.7264	0.08676	52 K.DSYVGDEAQSK.R 64
KRT2	P62736	Keratin, type II cytoskeletal 2 epidermal	1.8	4.91	0.00099	1191.8102	0.09092	374 K.YEELQVTVGR.H 385
				4.89	0.00012	1461.0514	0.45310	302 K.VDLLNQEIEFLK.V 315
				3.95	0.00025	1372.6575	0.48309	441 K.LNDLEEALQQAK.E 454
KRT1	P04264	Keratin, type II cytoskeletal 1	1.6	2.24	0.00899	993.8803	0.14940	443 K.LNDLEDALQQAK.E 456
				4.70	0.00027	1435.8816	1.01701	343 R.SLDLDSIIAEVK.A 356
				3.08	0.00072	2083.0051	0.17698	185 K.SLNNQFASFIDK.V 198
ALB	A6NBZ8	Uncharacterized protein ALB	1.6	2.95	0.04948	1444.1611	0.54524	286 K.YICENQDSISSK.L 299
				3.08	0.00704	1512.1173	0.04618	438 K.VPQVSTPTLVEVSR.N 453
				2.95	0.04947	1078.9591	0.10821	499 K.CCTESLVNR.R 509
**Down-regulated in VKH**								
SFRS1	Q07955	Isoform ASF-1 of Splicing factor, arginine/serine-rich 1	0.50	2.52	0.00007	1255.5932	0.37017	17 R.IYVGNLPPDIR.T 29
				2.78	0.03060	1078.9591	0.10822	154 R.DGTGVVEFVR.K 165
				3.37	0.00126	1417.7458	0.11825	142 R.EAGDVCYADVYR.D 155

a: average expression ratio;

b: cross-correlation score.

**Table 3:** The differently expressed proteins identified based on single peptides in CD4^+^ T cells between active VKH patients and normal individuals.

Genesymbol	Swiss-prot accession No.	Identified proteins	Expression Ratio^a^ (VKH:NC)	Xcorr^b^	t-test p	MH+(Da)	Mass stdev(Da)	Identified Peptide
**Up-regulated in VKH**								
								
ITGB2 (CD18)	A8MYE6	Integrin beta	2.9	3.66	0.00068	1047.7857	0.11764	520 R.TTEGCLNPR.R 530
ODZ3	Q9P273	Teneurin-3	2.5	4.03	0.00004	2254.3396	0.17867	897 R.QDGMFDLVANGGASLTLVFER.S 920
CORO2A	Q92828	Coronin-2A	2.4	2.21	0.00128	1550.6224	0.22761	235 K.KLMSTGTSRWNNR.Q 249
C19orf2	Q8TC23	C19orf2 protein	2.3	2.91	0.01076	1088.1358	0.47804	344 R.INTGKNTTLK.F 355
DMD	P11532	Isoform 4 of Dystrophin	2.1	4.05	0.00033	1226.9591	0.20370	602 K.LAVLKADLEKK.K 614
SYCP2	Q9BX26	Synaptonemal complex protein 2	2.1	3.18	0.00454	1427.8801	0.06260	509 R.IKPPLQMTSSAEK.P 523
DKFZp686D0972	Q562R1	hypothetical protein LOC345651	1.9	2.27	0.00140	1791.0197	0.11971	239 R.SYELPDGQVITIGNER.F 256
CDNA FLJ41329 fis, clone BRAMY2047676	Q6ZWC4	none	1.9	3.05	0.00548	1546.8087	0.08170	107 R.ALNLGAATVLRRHR.A 122
SLC22A11	Q9NSA0	Isoform 1 of Solute carrier family 22 member 11	1.9	2.63	0.00783	2210.0904	0.46292	301 R.INGHKEAKNLTIEVLMSSVK.E 322
RPS27A	P62979	UBC;UBB ubiquitin and ribosomal protein S27a precursor	1.8	3.39	0.01933	1788.1465	0.15637	11 K.TITLEVEPSDTIENVK.A 28
ARL6IP5	O75915	PRA1 family protein 3	1.8	2.85	0.00053	1312.8985	0.35814	9 R.AWDDFFPGSDR.F 21
ACTB	P60709	Actin, cytoplasmic 1	1.8	4.57	0.00029	1132.7391	0.08870	196 R.GYSFTTTAER.E 207
GPR179	A8MWI1	Probable G-protein coupled receptor 179 precursor	1.7	4.26	0.00002	1150.9204	0.12430	2099 R.GSSEAAGSVETR.V 2112
HSPA9	P38646	heat shock 70kDa protein 9, mitochondrial precursor	1.7	4.66	0.04236	1450.7788	0.13564	85 R.TTPSVVAFTADGER.L 100
FAM62A	Q9BSJ8	Isoform 1 of Protein FAM62A	1.6	2.68	0.02300	1402.3521	0.45065	106 R.QLLDDEEQLTAK.T 119
ELMO2	Q7Z5G9	ELMO2 protein	1.6	3.18	0.01234	1484.9090	0.09548	1 MERTQSSNMETR.L 13
CCDC73	Q6ZRK6	Isoform 1 of Coiled-coil domain-containing protein 73	1.6	2.39	0.01896	1583.8480	0.08212	84 K.EAMAVFKKQLQMK.M 98
**Down-regulated in VKH**								
PRPH	P41219	Isoform 1 of Peripherin	0.60	2.88	0.01008	1309.9330	0.17343	398 K.LLEGEESR.I 407
ATP5A1	P25705	ATP synthase subunit alpha, mitochondrial precursor	0.60	2.54	0.00232	1316.9060	0.22248	218 K.TSIAIDTIINQK.R 231
CANX	B4DGP8	Calnexin precursor	0.37	2.70	0.00003	1488.7949	0.25015	480 R.IVDDWANDGWGLK.K 494
AKNA	Q7Z591	Isoform 1 of AT-hook-containing transcription factor	0.33	3.75	0.00007	1236.9160	0.16416	330 R.PLPRQGATLAGR.S 342
ITGB2(CD18)	P05107	Integrin beta-2 precursor	0.24	2.54	0.00017	1090.0657	0.37385	155 R.ALNEITESGR.I 166

a: average expression ratio;

b: cross-correlation score.

### Validation of CD18 and AKNA by Western blot technique

**Figure 3:**
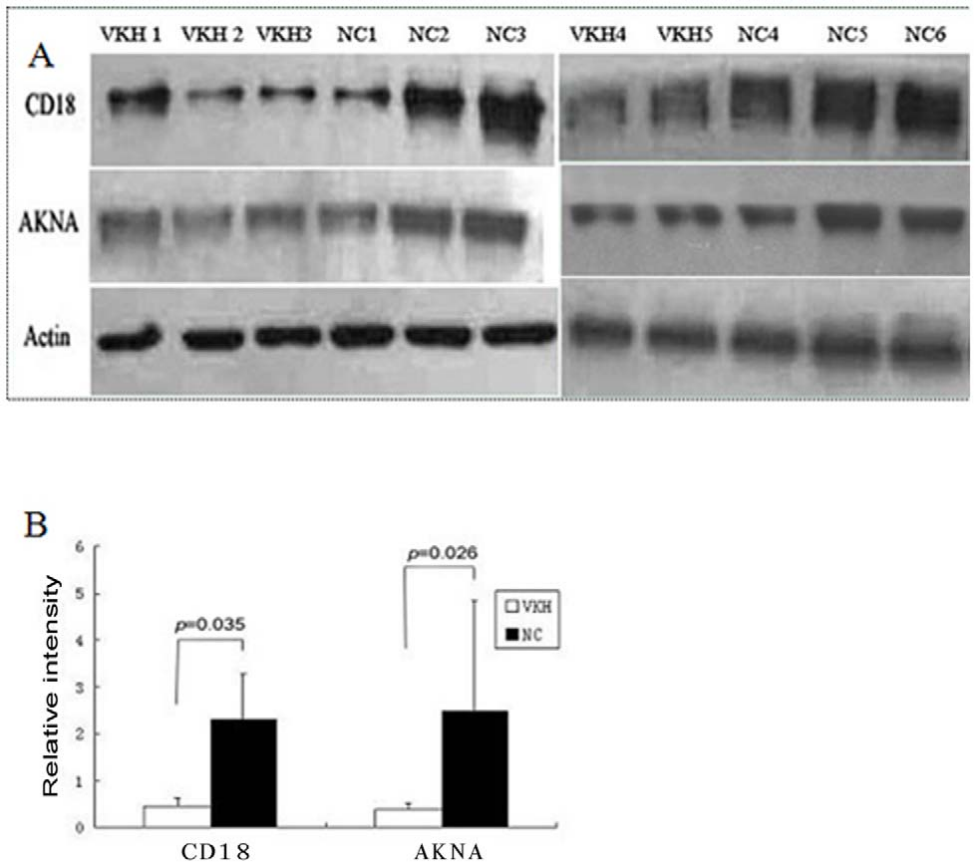
Validation of CD18 and AKNA by the Western blot technique. Antibodies were used at a dilution of 1∶1000 for the anti-human CD18 monoclonal antibody and anti-human AKNA monoclonal antibody. Proteins were detected using the Phototope-HRP Western blot detection system(A). The immunoreactive band intensities were quantitated and were presented as intensity volumes (vol%). The results showed that both CD18 and AKNA were significantly down-regulated in VKH patients as compared to normal controls (B). VKH: VKH patients, NC: normal controls.

Among the thirty proteins identified, two proteins, CD18 and AKNA, were found to have a more than three-fold difference between active VKH patients and normal controls. The different expression of CD18 in both samples was shown in magnified intensity graphs (Figure 2, C and D). Our study further validated the expression of these two proteins using the Western blot technique. The immunoreactive band intensities were quantitated and were presented as intensity volumes (vol %). The results showed that the expression of CD18 was about five-fold lower in the active VKH patients as compared to normal controls. As for the expression of AKNA, a similar result was observed (Figure 3). The results for both proteins were generally consistent with the quantitative data of the label-free method.

## Discussion

In this study, label-free relative quantitative proteomics was employed to screen differently expressed membrane proteins in CD4^+^ T cells from active VKH patients and normal controls. Thirty proteins were identified to be at least 1.5 fold differently expressed between active VKH patients and normal individuals. A significantly decreased expression of CD18 and AKNA was further confirmed using the Western blot technique. The results presented here may provide new clues for the study on molecules involved in the pathogenesis of VKH syndrome.

CD4^+^ T cells have been shown to be crucial in the pathogenesis of this syndrome [5], [6], [22]. The present study aimed at searching for the differently expressed proteins in these cells between active VKH patients and normal controls. Proteomics has provided useful strategies for the screening of differently expressed proteins. The recently established label-free strategy has been proven to be more efficient as compared to conventional 2-DE approaches [18], [19], [20], [21]. It has been used to identify urinary biomarkers from antineutrophil cytoplasmic antibodies [23] and differently expressed proteins from patients with ductal carcinoma [24] and Parkinson's disease [25]. These studies have proven that the label-free strategy is a powerful tool for identifying disease-associated proteins. In this study, we employed the label-free approach to identify differently expressed proteins in CD4+ T cells, an important population involved in various autoimmune diseases, between active VKH patients and normal controls.

To ensure the accuracy and stability of the label-free quantitative analysis, several procedures were performed. For the purpose of minimizing the effect of individual differences on the experimental results, an equal amount of membrane proteins of CD4+ T cells from five active VKH patients and five normal controls were pooled respectively and subjected to the label-free quantitative proteomics. For label-free proteomics, strategies using three or four technical replicates have been reported [16], [17], [26]. As three technical replicates are a minimal requirement for statistical analysis and have already been justified by a number of studies, we chose three technical replicates in the present study. Our results showed a great reproducibility in peptide quantitation (Table S1). To acquire stable MS data needed for quantitative analysis, several quality control measures were used [16]. The data from each run of both VKH patients and normal controls appeared to be reproducible in terms of mass accuracy, retention time and intensity. Binary comparison maps used in the experiment validated the reproducibility between duplicates. Coefficient of variation (CV) analysis [17] was performed and the result showed that the average CV of the fold changes was 23% for proteins with two or more peptides, which ensured the accuracy of protein quantitation (Table S2).

Our label-free proteomic results showed that thirty proteins were different in expression for more than 1.5 fold between active VKH patients and normal controls,although these results need to be confirmed by other techniques. By searching for the functions of these proteins according to annotations in the Swiss-prot database at http://expasy.org, we interestingly noted that these identified proteins are involved in 54 biological processes (Table S3) and 39 functional groups (Table S4). Importantly, we found that a number of molecules, including vimentin, dystrophin, Keratin 10, AKNA and CD18, are associated with an inflammatory and/or immune response. An abnormal expression of certain proteins has also been found to be involved in autoimmune diseases or immune related diseases [27], [28], [29]. Enhanced vimentin immunoreactivity was found in the spinal cord of rats with experimental autoimmune encephalomyelitis [28]. Increased expression of dystrophin was observed in experimental autoimmune myasthenia gravis [27].

By analyzing the data acquired using the label-free method, it was interesting to note that a number of proteins identified in this study, including vimentin and AKNA transcription factor, were non-membrane components. A similar result has also been observed in proteomic studies. These non-membrane molecules could be explained as being a component associated with the membrane or with membrane fractions [30], [31]. It is worthy to point out that the increased expression of keratins may be involved in the pathogenesis of a number of diseases affecting the skin [32], [33], [34], [35]. It is not clear whether keratins are truly induced and, if it is, how it functions in VKH syndrome, an autoimmune disease frequently affecting the integumentary system.

False positive data always exist in proteomic studies. Therefore, the proteomic results usually need to be confirmed using other techniques. As protein with a big different expression ratio is readily confirmed by another technique, we chose two proteins with a three-fold different expression between VKH patients and normal controls, CD18 and AKNA, as candidates in the Westernblot identification. As expected, the Western blot result revealed a five-fold lower expression of both proteins in active VKH patients as compared with normal controls. It is worthwhile to point out that both CD18 and AKNA were identified using the one peptide based identification, as already reported previously [36], [37]. This could be due to the lower abundance of both proteins in the tested samples.

CD18, also known as integrin beta 2 or ITGB2, is one of the members of the integrin family, which is involved in cell adhesion, neutrophil chemotaxis, and cell-surface mediated signaling. The expression of CD18 can be down-regulated by introducing an insertion mutation in the murine CD18 gene. Due to this mutation, a chronic inflammatory skin disease clinically resembling human psoriasis develops in PL/J mice [38], [39]. It has been reported that resistance of T cells to apoptosis may be involved in the pathogenesis or perpetuation of VKH syndrome [5], [6]. CD18 has been shown to be required in apoptosis of human T cells [40], [41], [42]. A decreased expression of CD18 in active VKH patients may suggest that an insufficient inhibition on apoptosis of CD4^+^ T cells is present at the active stage.

ANKA, also known as AHCTF1, is reported to regulate the expression of CD40 and CD40L [43], both molecules essential in immune and inflammatory responses. The interaction of CD40 and CD40L has been shown to be involved in the negative regulation of T cell autoreactivity and abnormalities in their interaction may lead to autoimmunity. In the present study, a significantly decreased expression of AKNA was noted in CD4^+^ T cells from active VKH patients. The decreased AKNA may be presumed to down-regulate the expression of CD40L in CD4^+^ T cells. Such a decreased expression of CD40L has indeed been observed in peripheral CD4^+^ T cells in autoimmune thyroid disease [44]. More studies are needed to clarify whether a decreased AKNA could play a role in VKH syndrome via down-regulating CD40 and CD40L.

In summary, label-free quantitative proteomics showed abnormal expression of thirty proteins for more than 1.5 fold in active VKH patients. A significantly decreased expression of CD18 and AKNA was confirmed using the Western blot technique. Both proteins may be involved in the pathogenesis of VKH syndrome. However, our study did not elucidate the mechanism by which both proteins exert their function. Further studies about the effect of both proteins on cytokine expression, apoptosis and chemotaxis of CD4^+^ T cells will contribute to our understanding about the mechanisms in the pathogenesis of VKH syndrome.

## Supporting Information

Figure S1 A representative binary comparison map of duplicate runs of samples from VKH patients. The horizontal (x) axis represents the distribution of peak intensities of the first run, and the vertical (y) axis represents that of the second run. The average intensity correlation coefficient (CC) between the two runs was 0.92. The expected distribution of the duplicate runs showed no obvious change.
(0.04 MB PDF)

Figure S2 A representative base peak ion chromatogram from LC-MS/MS analysis of a total membrane protein extract from the VKH group digested with trypsin.
(0.19 MB PDF)

Figure S3 The MS/MS spectra for single-peptide-based identifications. The detected b and y ions used in the protein identification were labeled in the MS/MS spectra. The unlabeled peaks are a, c, x or z ions generated in mass spectrometer.
(1.88 MB PDF)

Table S1 All the identified peptides and their sequences of each protein in both samples.
(0.08 MB XLS)

Table S2 C.V.analysis of all the identified proteins based on at least two peptides.The average CV of the fold changes was 23% for proteins with two or more peptides.
(0.06 MB XLS)

Table S3 Biological Process of the identified proteins.
(0.02 MB XLS)

Table S4 The molecular function of the identified proteins.
(0.08 MB XLS)
