# Correction: Label-Free Proteomics Reveals Decreased Expression of CD18 and AKNA in Peripheral CD4^+^ T Cells from Patients with Vogt-Koyanagi-Harada Syndrome

**DOI:** 10.1371/annotation/2c8e3e74-556c-4e42-a5df-0b8ce294119f

**Published:** 2011-03-04

**Authors:** Liming Mao, Peizeng Yang, Shengping Hou, Fuzhen Li, Aize Kijlstra

The first author affiliation is incorrect. It should read "The First Affiliated Hospital of Chongqing Medical University, Chongqing Key Laboratory of Ophthalmology, Chongqing Eye Institute, Chongqing, People&rsquo;s Republic of China." 

