# Supplementary material for: Label-Free Proteomics Reveals Decreased Expression of CD18 and AKNA in Peripheral CD4+ T Cells from Patients with Vogt-Koyanagi-Harada Syndrome
Source: PLoS One. 2011 Jan 28;6(1):e14616. doi: 10.1371/journal.pone.0014616 (PMC3030555; doi:10.1371/journal.pone.0014616)
Supplement: Figure S1 — A representative binary comparison map of duplicate runs of samples from VKH patients. The horizontal (x) axis represents the distribution of peak intensities of the first run, and the vertical (y) axis represents that of the second run. The average intensity correlation coefficient (CC) between the two runs was 0.92. The expected distribution of the duplicate runs showed no obvious change. (0.04 MB PDF) [file pone.0014616.s001.pdf]

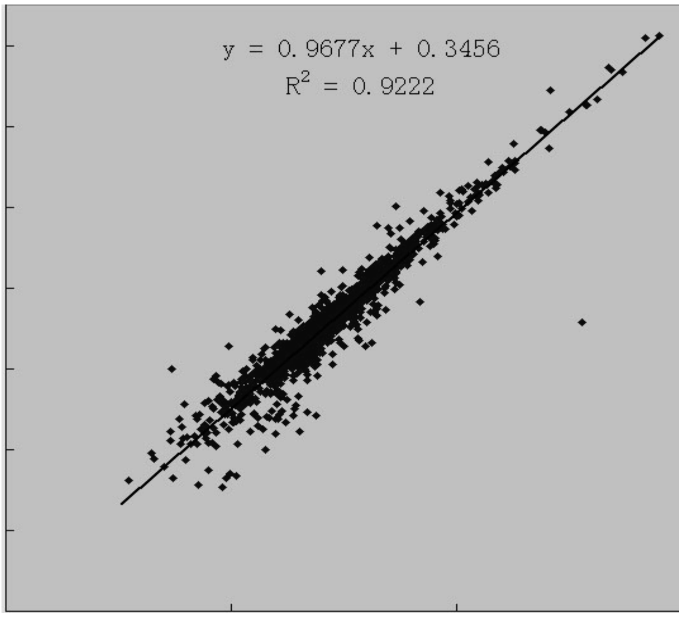

Figure S1

A representative binary comparison map of duplicate runs of samples from VKH patients. The horizontal (x) axis represents the distribution of peak intensities of the first run, and the vertical (y) axis represents that of the second run. The average intensity correlation coefficient (CC) between the two runs was 0.92. The expected distribution of the duplicate runs showed no obvious change.
