# Supplementary material for: Label-Free Proteomics Reveals Decreased Expression of CD18 and AKNA in Peripheral CD4+ T Cells from Patients with Vogt-Koyanagi-Harada Syndrome
Source: PLoS One. 2011 Jan 28;6(1):e14616. doi: 10.1371/journal.pone.0014616 (PMC3030555; doi:10.1371/journal.pone.0014616)
Supplement: Figure S2 — A representative base peak ion chromatogram from LC-MS/MS analysis of a total membrane protein extract from the VKH group digested with trypsin. (0.19 MB PDF) [file pone.0014616.s002.pdf]

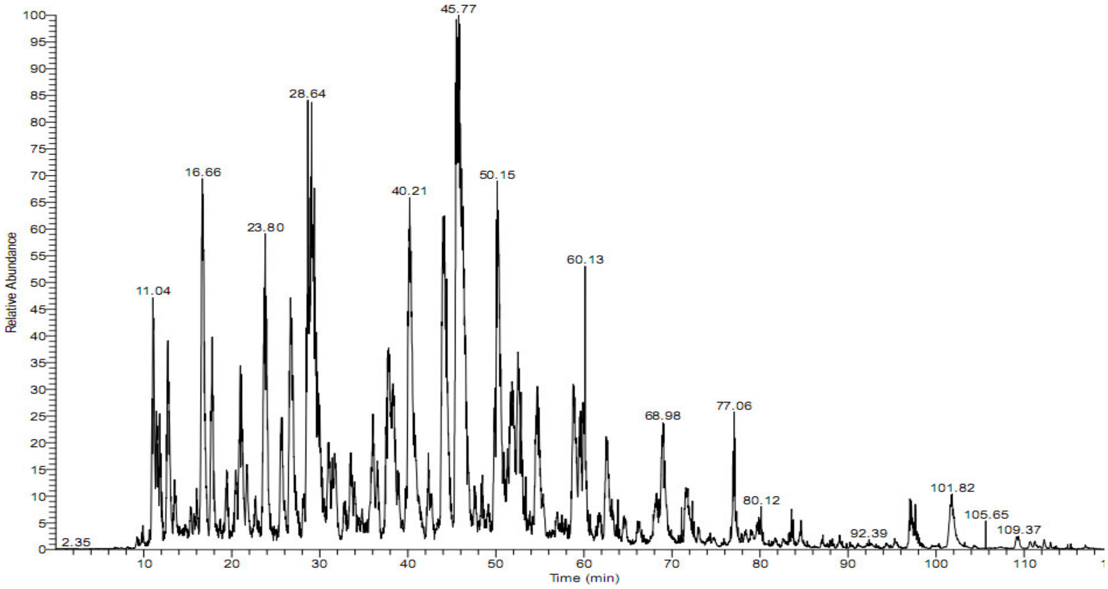


**Figure S2**

**A representative base peak ion chromatogram from LC-MS/MS analysis of a total membrane protein extract from the VKH group digested with trypsin.**
