# Supplementary material for: Label-Free Proteomics Reveals Decreased Expression of CD18 and AKNA in Peripheral CD4+ T Cells from Patients with Vogt-Koyanagi-Harada Syndrome
Source: PLoS One. 2011 Jan 28;6(1):e14616. doi: 10.1371/journal.pone.0014616 (PMC3030555; doi:10.1371/journal.pone.0014616)
Supplement: Figure S3 — The MS/MS spectra for single-peptide-based identifications. The detected b and y ions used in the protein identification were labeled in the MS/MS spectra. The unlabeled peaks are a, c, x or z ions generated in mass spectrometer. (1.88 MB PDF) [file pone.0014616.s003.pdf]

A8MYE6 **Integrin beta** MH+ (Da): 1047.7857

b ions			y ions		
b1	T	102.1			
b2	TT	**203.2**	y9	TTEGCLNPR	991.1
b3	TTE	**332.3**	y8	TEGCLNPR	890
b4	TTEG	**389.39**	y7	EGCLNPR	788.9
b5	TTEGC	492.53	y6	GCLNPR	**659.8**
b6	TTEGCL	**605.69**	y5	CLNPR	602.7
b7	TTEGCLN	**719.79**	y4	LNPR	**499.6**
b8	TTEGCLNP	816.91	y3	NPR	**386.4**
b9	TTEGCLNPR	973.1	y2	PR	**272.3**
			y1	R	175.2

The detected ions were shown in bold.

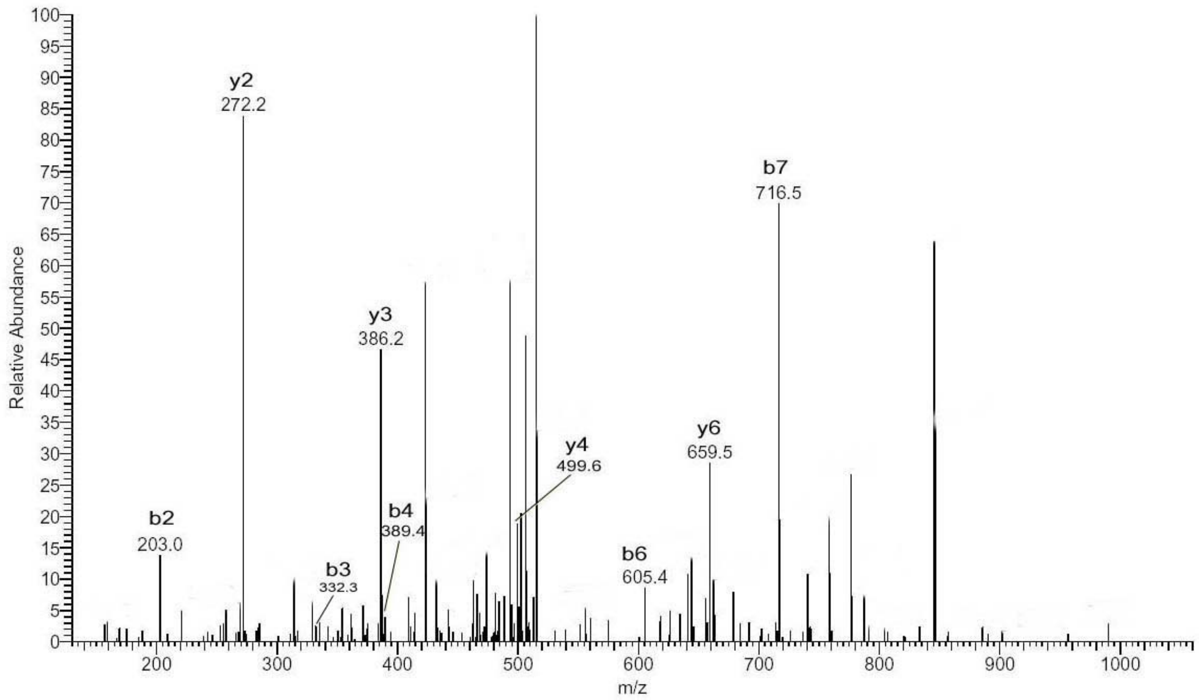

Q9P273	**Teneurin-3**		MH+ (Da):	2254.3396	
b ions			y ions		
b1	Q	129.15			
b2	QD	244.23	y21	QDGMFDLVANGGASLTLVFER	2257.52
b3	QDG	301.29	y20	DGMFDLVANGGASLTLVFER	2129.39
b4	QDGM	448.48	y19	GMFDLVANGGASLTLVFER	2014.3
b5	QDGMF	595.66	y18	MFDLVANGGASLTLVFER	1957.24
b6	QDGMFD	710.74	y17	FDLVANGGASLTLVFER	**1810.05**
b7	QDGMFDL	823.9	y16	DLVANGGASLTLVFER	**1662.88**
b8	QDGMFDLV	923.04	y15	LVANGGASLTLVFER	**1547.79**
b9	QDGMFDLVA	994.12	y14	VANGGASLTLVFER	1434.63
b10	QDGMFDLVAN	1108.22	y13	ANGGASLTLVFER	1335.49
b11	QDGMFDLVANG	1165.27	y12	NGGASLTLVFER	1264.42
b12	QDGMFDLVANGG	**1222.32**	y11	GGASLTLVFER	1150.31
b13	QDGMFDLVANGGA	1293.4	y10	GASLTLVFER	**1093.26**
b14	QDGMFDLVANGGAS	1380.48	y9	ASLTLVFER	1036.21
b15	QDGMFDLVANGGASL	1493.64	y8	SLTLVFER	**965.13**
b16	QDGMFDLVANGGASLT	1594.74	y7	LTLVFER	**878.05**
b17	QDGMFDLVANGGASLTL	1707.9	y6	TLVFER	**764.89**
b18	QDGMFDLVANGGASLTLV	1807.04	y5	LVFER	663.79
b19	QDGMFDLVANGGASLTLVF	1954.21	y4	VFER	550.63
b20	QDGMFDLVANGGASLTLVFE	2083.33	y3	FER	451.49
b21	QDGMFDLVANGGASLTLVFER	2239.52	y2	ER	304.32
			y1	R	175.2

The detected ions were shown in bold.

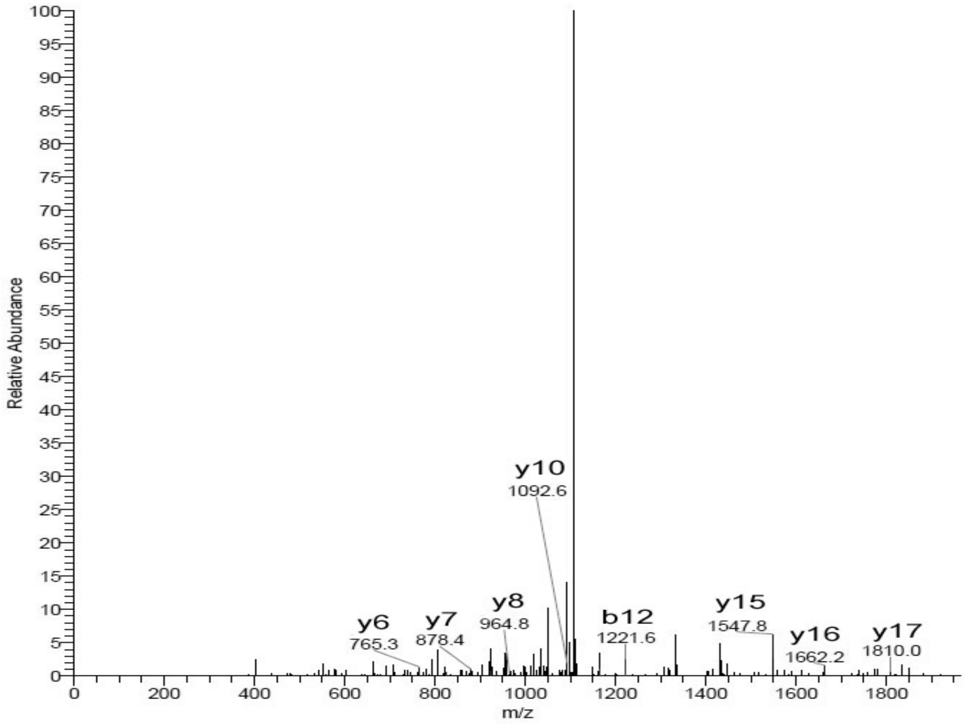

Q92828 **Coronin-2A** MH+ (Da)： 1550.6224

b ions			y ions		
b1	K	129. 19	y13	KLMSTGTSRWNNR	1551. 76
b2	KL	**242. 35**	y12	LMSTGTSRWNNR	1423. 58
b3	KLM	**373. 54**	y11	MSTGTSRWNNR	1310. 42
b4	KLMS	**460. 62**	y10	STGTSRWNNR	1179. 23
b5	KLMST	**561. 72**	y9	TGTSRWNNR	**1092. 15**
b6	KLMSTG	618. 78	y8	GTSRWNNR	**991. 05**
b7	KLMSTGT	**719. 88**	y7	TSRWNNR	**933. 99**
b8	KLMSTGTS	**806. 96**	y6	SRWNNR	**832. 89**
b9	KLMSTGTSR	**963. 15**	y5	RWNNR	**745. 81**
b10	KLMSTGTSRW	1149. 36	y4	WNNR	589. 62
b11	KLMSTGTSRWN	1263. 46	y3	NNR	**403. 41**
b12	KLMSTGTSRWNN	1377. 57	y2	NR	**289. 31**
b13	KLMSTGTSRWNNR	1533. 76	y1	R	175. 2

The detected ions were shown in bold.

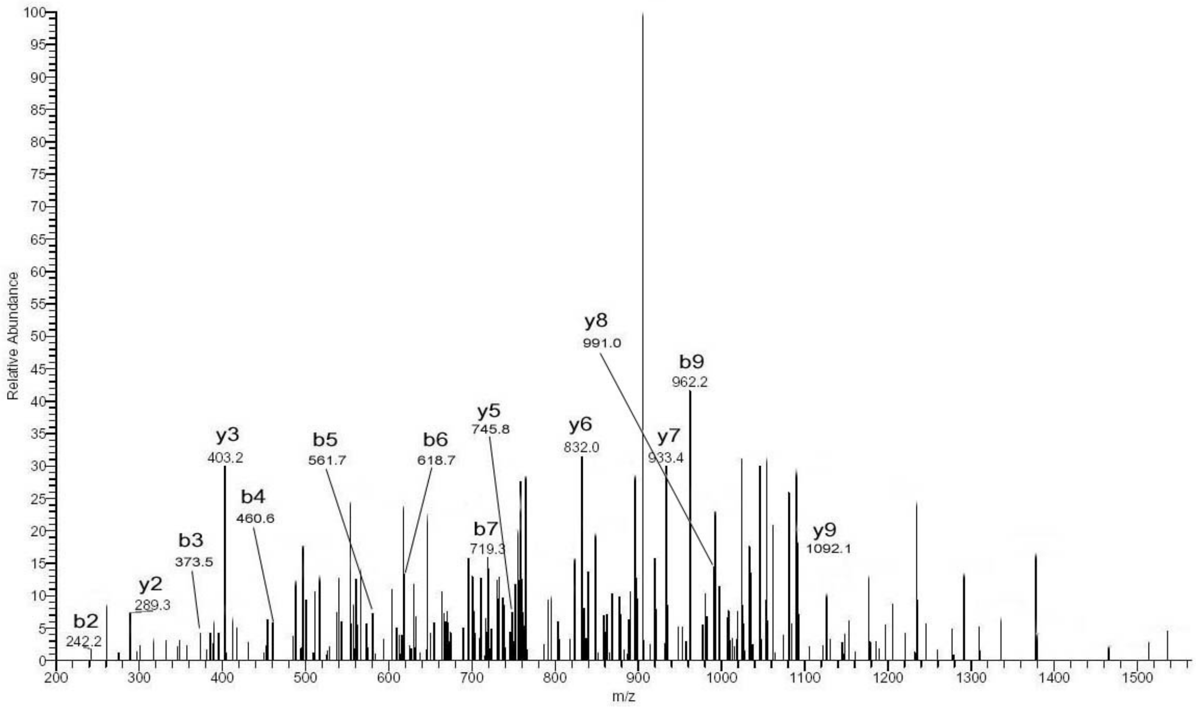

Q8TC23 **C19orf2 protein** MH+ (Da)： 1088.1358

b ions y ions

b1	I	114. 17			
b2	IN	228. 28	y10	INTGKNTTLK	1090. 26
b3	INT	329. 38	y9	NTGKNTTLK	977. 1
b4	INTG	**386. 44**	y8	TGKNTTLK	862. 99
b5	INTGK	**514. 61**	y7	GKNTTLK	761. 89
b6	INTGKN	**628. 71**	y6	KNTTLK	**704. 84**
b7	INTGKNT	**729. 82**	y5	NTTLK	**576. 66**
b8	INTGKNTT	830. 92	y4	TTLK	**462. 56**
b9	INTGKNTTL	**944. 08**	y3	TLK	**361. 45**
b10	INTGKNTTLK	**1072. 26**	y2	LK	260. 35
			y1	K	147. 19

The detected ions were shown in bold.

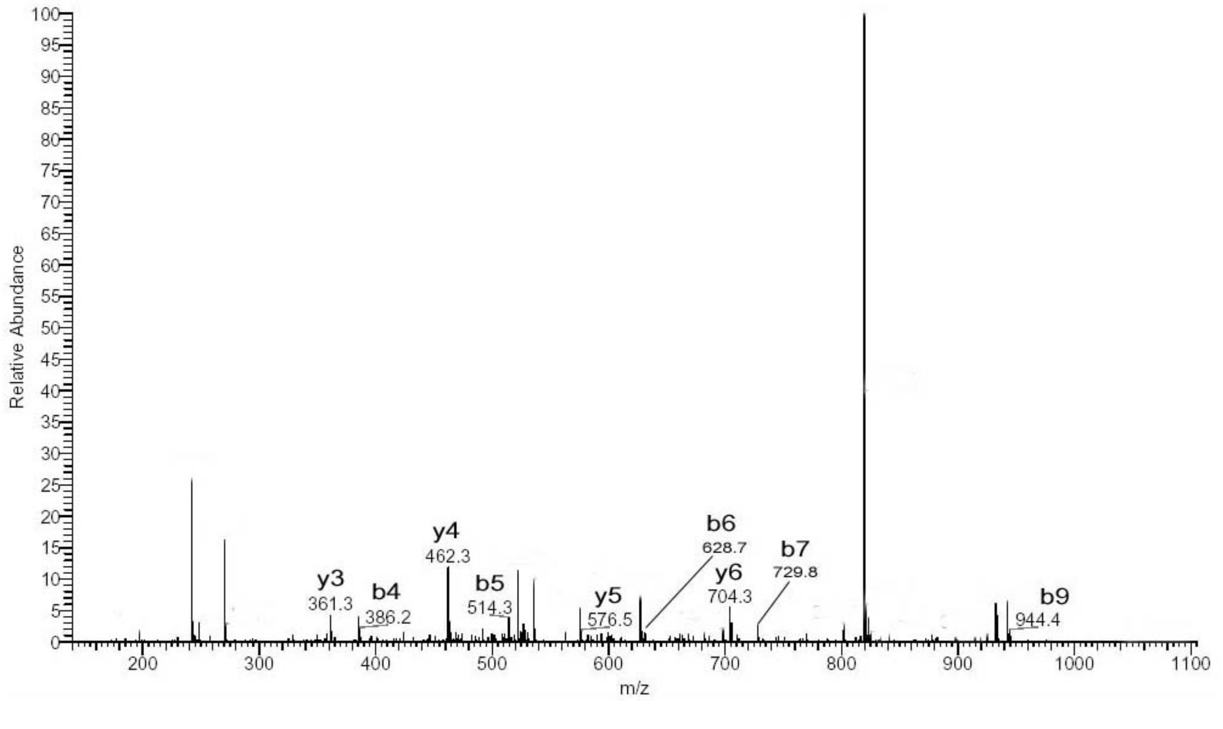

P11532 b ions	Isoform 4 of Dystrophin		MH+ (Da): y ions	1226.9591	
b1	L	114.17	y11	LAVLKADLEKK	1227.51
b2	LA	185.25	y10	AVLKADLEKK	**1114.35**
b3	LAV	**284.39**	y9	VLKADLEKK	1043.27
b4	LAVL	**397.55**	y8	LKADLEKK	**944.14**
b5	LAVLK	525.72	y7	KADLEKK	**830.98**
b6	LAVLKA	**596.8**	y6	ADLEKK	702.81
b7	LAVLKAD	**711.89**	y5	DLEKK	631.73
b8	LAVLKADL	825.05	y4	LEKK	516.64
b9	LAVLKADLE	954.16	y3	EKK	403.48
b10	LAVLKADLEK	**1082.34**	y2	KK	**274.36**
b11	LAVLKADLEKK	1210.51	y1	K	146.19

The detected ions were shown in bold.

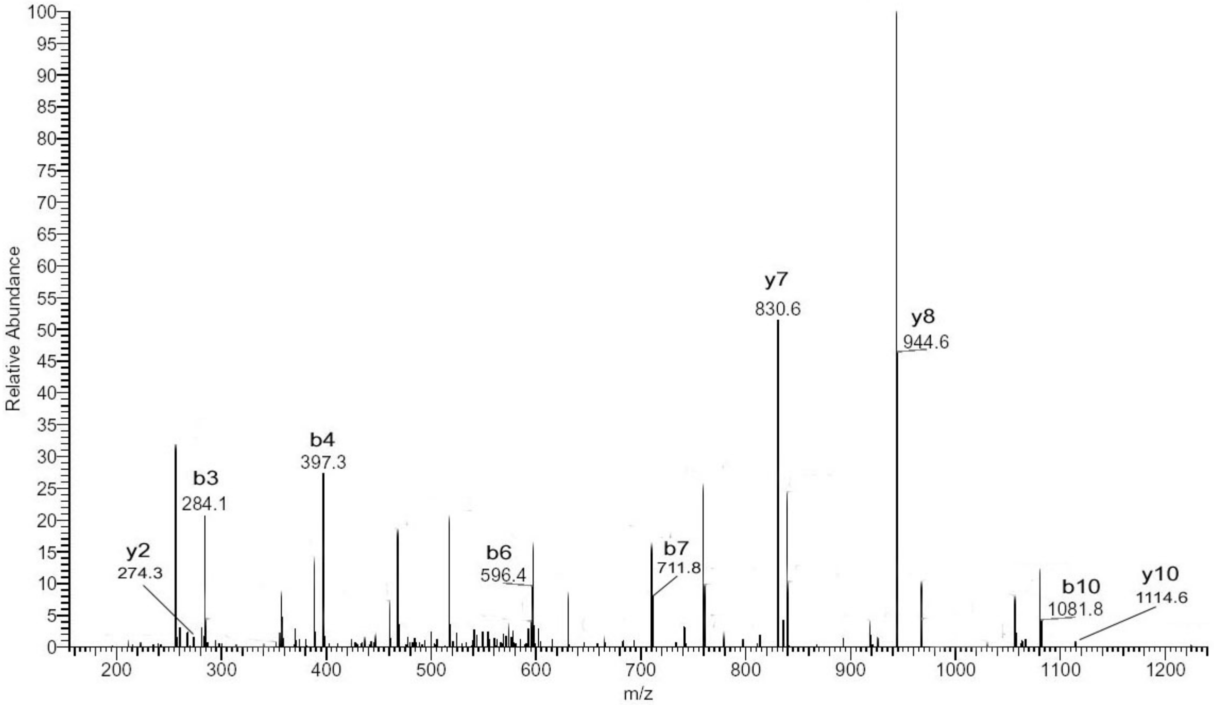

Q9BX26	**Synaptonemal complex protein 2**		MH+ (Da):	1427.8801	
b ions			y ions		
b1	I	114.17	y13	IKPPLQMTSSAEK	1430.69
b2	IK	242.35	y12	KPPLQMTSSAEK	1317.54
b3	IKP	**339.47**	y11	PPLQMTSSAEK	1189.36
b4	IKPP	436.58	y10	PLQMTSSAEK	1092.24
b5	IKPPL	**549.74**	y9	LQMTSSAEK	**995.13**
b6	IKPPLQ	**677.87**	y8	QMTSSAEK	881.97
b7	IKPPLQM	**809.06**	y7	MTSSAEK	753.84
b8	IKPPLQMT	**910.17**	y6	TSSAEK	**622.65**
b9	IKPPLQMTS	997.25	y5	SSAEK	**521.54**
b10	IKPPLQMTSS	**1084.33**	y4	SAEK	**434.46**
b11	IKPPLQMTSSA	1155.41	y3	AEK	347.38
b12	IKPPLQMTSSAE	1284.52	y2	EK	**276.3**
b13	IKPPLQMTSSAEK	1412.69	y1	K	147.19

The detected ions were shown in bold.

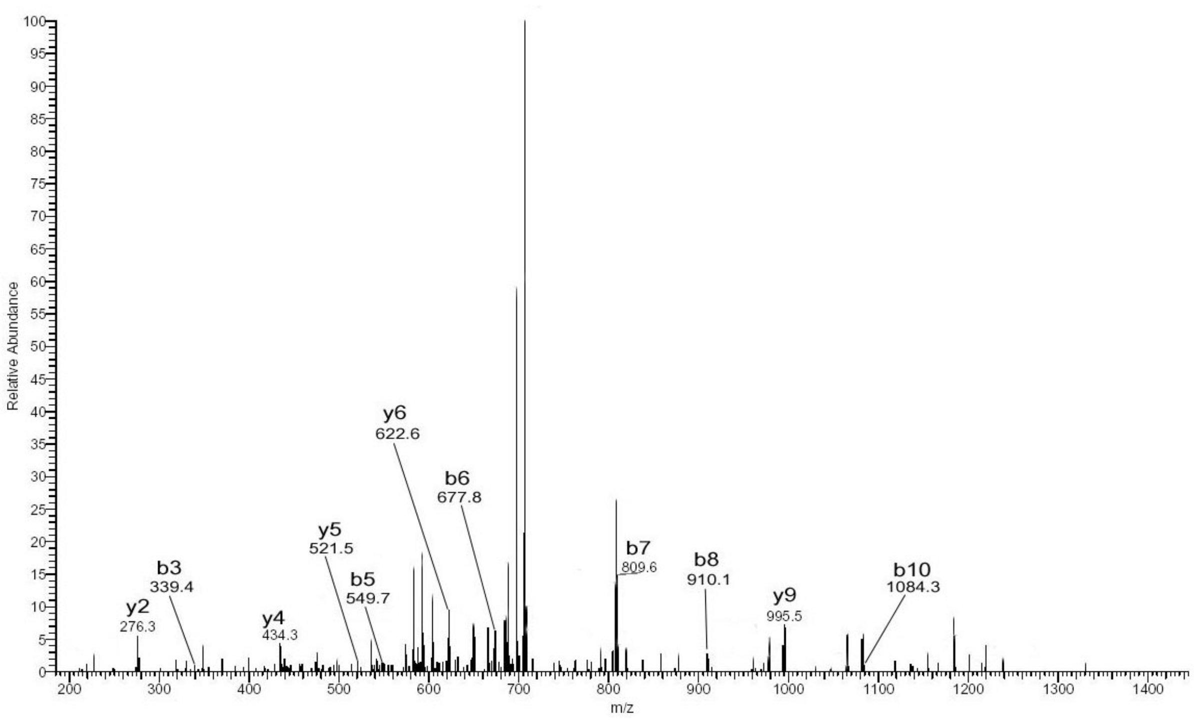

Q562R1	**hypothetical protein LOC345651**		MH+ (Da)：	1791.0197	
b ions			y ions		
b1	S	88.09	y16	SYELPDGQVITIGNER	1791.95
b2	SY	251.27	y15	YELPDGQVITIGNER	1704.87
b3	SYE	**380.38**	y14	ELPDGQVITIGNER	1541.69
b4	SYEL	**493.54**	y13	LPDGQVITIGNER	**1412.58**
b5	SYELP	**590.66**	y12	PDGQVITIGNER	**1299.42**
b6	SYELPD	705.75	y11	DGQVITIGNER	1202.3
b7	SYELPDG	762.8	y10	GQVITIGNER	1087.21
b8	SYELPDGQ	890.93	y9	QVITIGNER	**1030.16**
b9	SYELPDGQV	**990.06**	y8	VITIGNER	902.03
b10	SYELPDGQVI	**1103.22**	y7	ITIGNER	**802.9**
b11	SYELPDGQVIT	**1204.33**	y6	TIGNER	**689.74**
b12	SYELPDGQVITI	**1317.49**	y5	IGNER	**588.63**
b13	SYELPDGQVITIG	**1374.54**	y4	GNER	**475.47**
b14	SYELPDGQVITIGN	1488.64	y3	NER	**418.42**
b15	SYELPDGQVITIGNE	1617.76	y2	ER	304.32
b16	SYELPDGQVITIGNER	1773.95	y1	R	175.2

The detected ions were shown in bold.

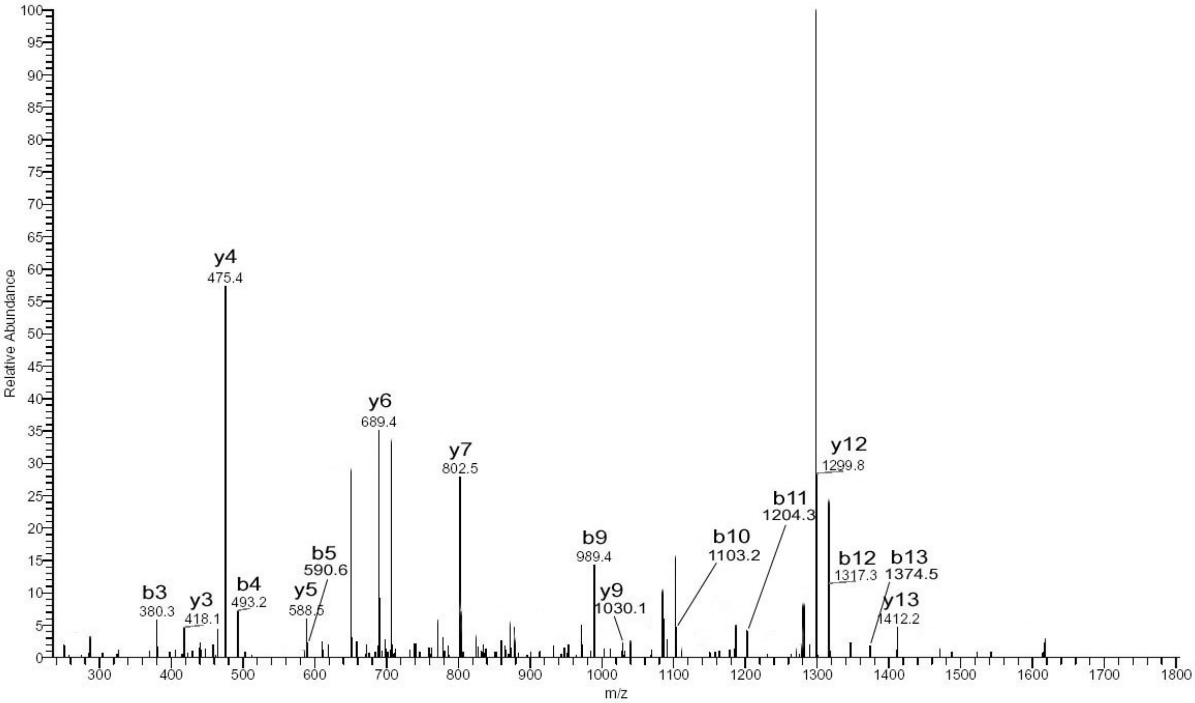

Q6ZWC4	**none**		MH+ (Da):	1546.8087	
b ions			y ions		
b1	A	72.09	y14	ALNLGAATVLRRHR	1548.83
b2	AL	185.25	y13	LNLGAATVLRRHR	1477.75
b3	ALN	299.36	y12	NLGAATVLRRHR	**1364.59**
b4	ALNL	412.52	y11	LGAATVLRRHR	**1250.48**
b5	ALNLG	469.57	y10	GAATVLRRHR	1137.33
b6	ALNLGA	540.65	y9	AATVLRRHR	**1080.27**
b7	ALNLGAA	**611.73**	y8	ATVLRRHR	1009.19
b8	ALNLGAAT	**712.83**	y7	TVLRRHR	**938.12**
b9	ALNLGAATV	**811.96**	y6	VLRRHR	**837.01**
b10	ALNLGAATVL	925.12	y5	LRRHR	737.88
b11	ALNLGAATVLR	**1081.31**	y4	RRHR	**624.72**
b12	ALNLGAATVLRR	1237.5	y3	RHR	468.53
b13	ALNLGAATVLRRH	**1374.64**	y2	HR	312.34
b14	ALNLGAATVLRRHR	1530.83	y1	R	175.2

The detected ions were shown in bold.

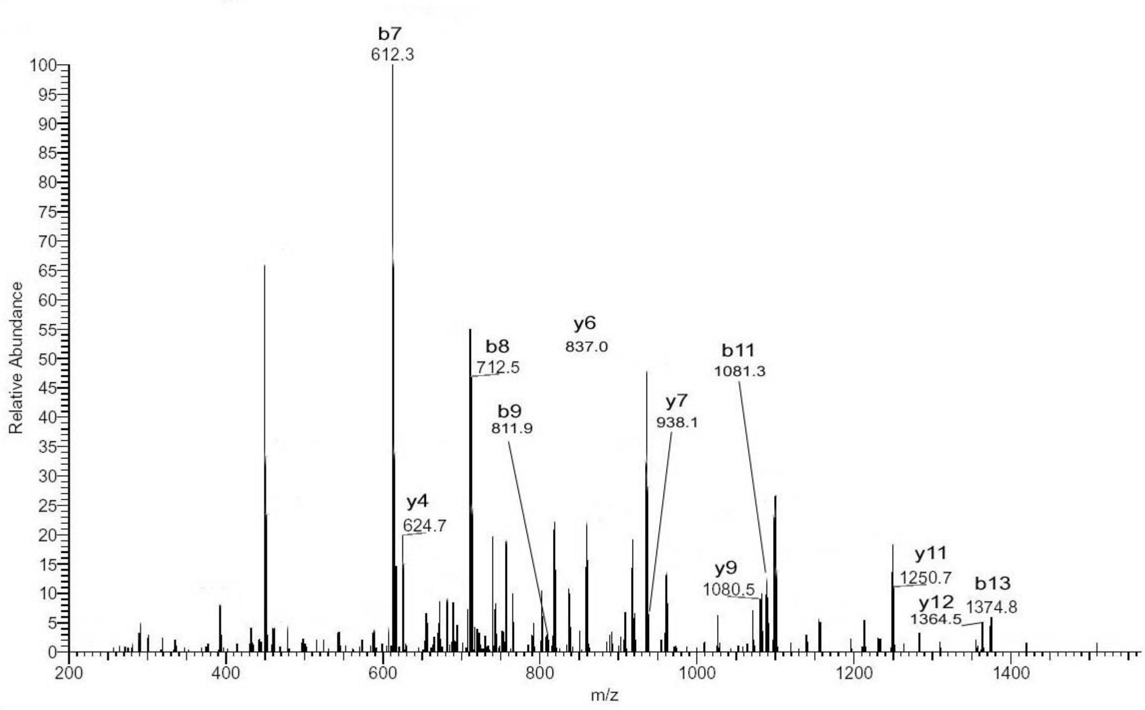

Q9NSA0	Isoform 1 of Solute carrier family 22 member 11		MH+ (Da):	2210.0904	
b ions			y ions		
b1	I	114.17	y20	INGHKEAKNLTIEVLMSSVK	2212.6
b2	IN	228.28	y19	NGHKEAKNLTIEVLMSSVK	2099.45
b3	ING	285.33	y18	GHKEAKNLTIEVLMSSVK	1985.34
b4	INGH	422.47	y17	HKEAKNLTIEVLMSSVK	1928.29
b5	INGHK	**550.65**	y16	KEAKNLTIEVLMSSVK	**1791.15**
b6	INGHKE	679.76	y15	EAKNLTIEVLMSSVK	**1662.97**
b7	INGHKEA	750.84	y14	AKNLTIEVLMSSVK	1533.86
b8	INGHKEAK	**879.01**	y13	KNLTIEVLMSSVK	1462.78
b9	INGHKEAKN	993.12	y12	NLTIEVLMSSVK	**1334.61**
b10	INGHKEAKNL	1106.28	y11	LTIEVLMSSVK	1220.5
b11	INGHKEAKNLT	1207.38	y10	TIEVLMSSVK	1107.34
b12	INGHKEAKNLTI	1320.54	y9	IEVLMSSVK	1006.24
b13	INGHKEAKNLTIE	1449.66	y8	EVLMSSVK	893.08
b14	INGHKEAKNLTIEV	**1548.79**	y7	VLMSSVK	763.96
b15	INGHKEAKNLTIEVL	**1661.95**	y6	LMSSVK	**664.83**
b16	INGHKEAKNLTIEVLM	**1793.14**	y5	MSSVK	**551.67**
b17	INGHKEAKNLTIEVLMS	1880.22	y4	SSVK	420.48
b18	INGHKEAKNLTIEVLMSS	1967.3	y3	SVK	333.4
b19	INGHKEAKNLTIEVLMSSV	2066.43	y2	VK	246.32
b20	INGHKEAKNLTIEVLMSSVK	2194.6	y1	K	147.19

The detected ions were shown in bold.

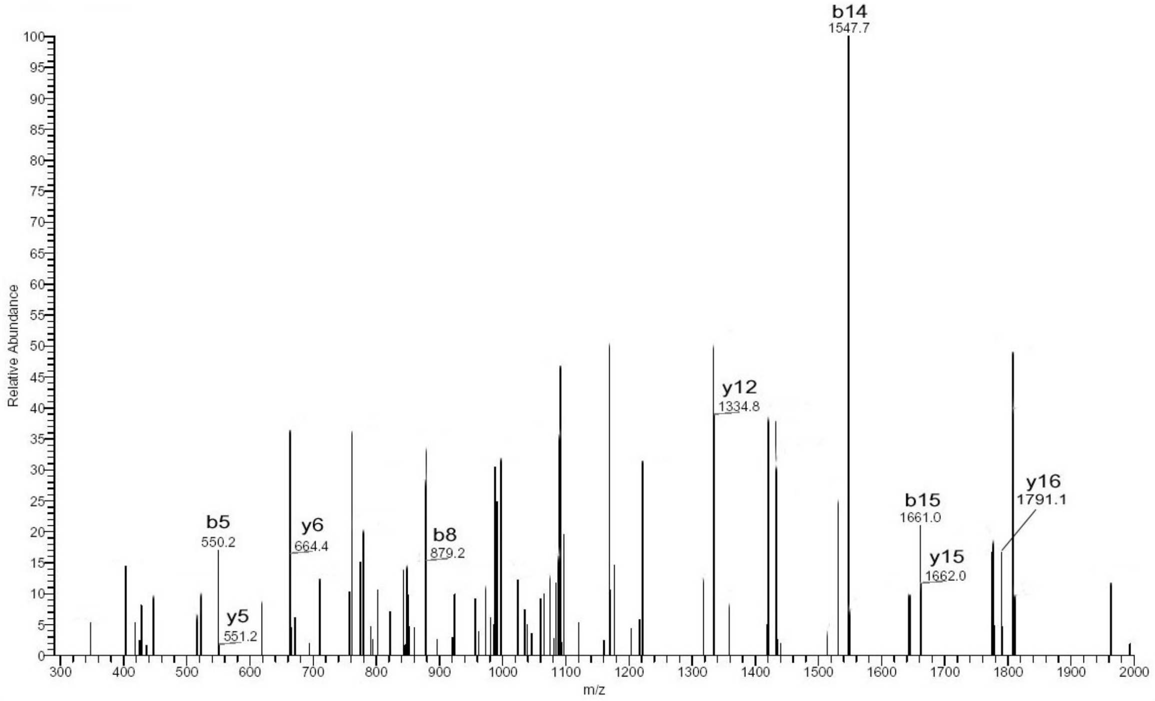

P62979	**UBC;UBB ubiquitin and ribosomal protein S27a precursor**		MH+ (Da):	1788.1465	
b ions			y ions		
b1	T	102.12	y16	TITLEVEPSDTIENVK	1788.98
b2	TI	215.28	y15	ITLEVEPSDTIENVK	1687.88
b3	TIT	316.38	y14	TLEVEPSDTIENVK	1574.72
b4	TITL	**429.54**	y13	LEVEPSDTIENVK	1473.61
b5	TITLE	558.66	y12	EVEPSDTIENVK	**1360.45**
b6	TITLEV	**657.79**	y11	VEPSDTIENVK	**1231.34**
b7	TITLEVE	**786.91**	y10	EPSDTIENVK	**1132.2**
b8	TITLEVEP	**884.02**	y9	PSDTIENVK	**1003.09**
b9	TITLEVEPS	971.1	y8	SDTIENVK	905.97
b10	TITLEVEPSD	1086.19	y7	DTIENVK	818.89
b11	TITLEVEPSDT	1187.3	y6	TIENVK	703.81
b12	TITLEVEPSDTI	1300.46	y5	IENVK	602.7
b13	TITLEVEPSDTIE	**1429.57**	y4	ENVK	**489.54**
b14	TITLEVEPSDTIEN	**1543.68**	y3	NVK	**360.43**
b15	TITLEVEPSDTIENV	**1642.81**	y2	VK	246.32
b16	TITLEVEPSDTIENVK	1770.98	y1	K	147.19

The detected ions were shown in bold.

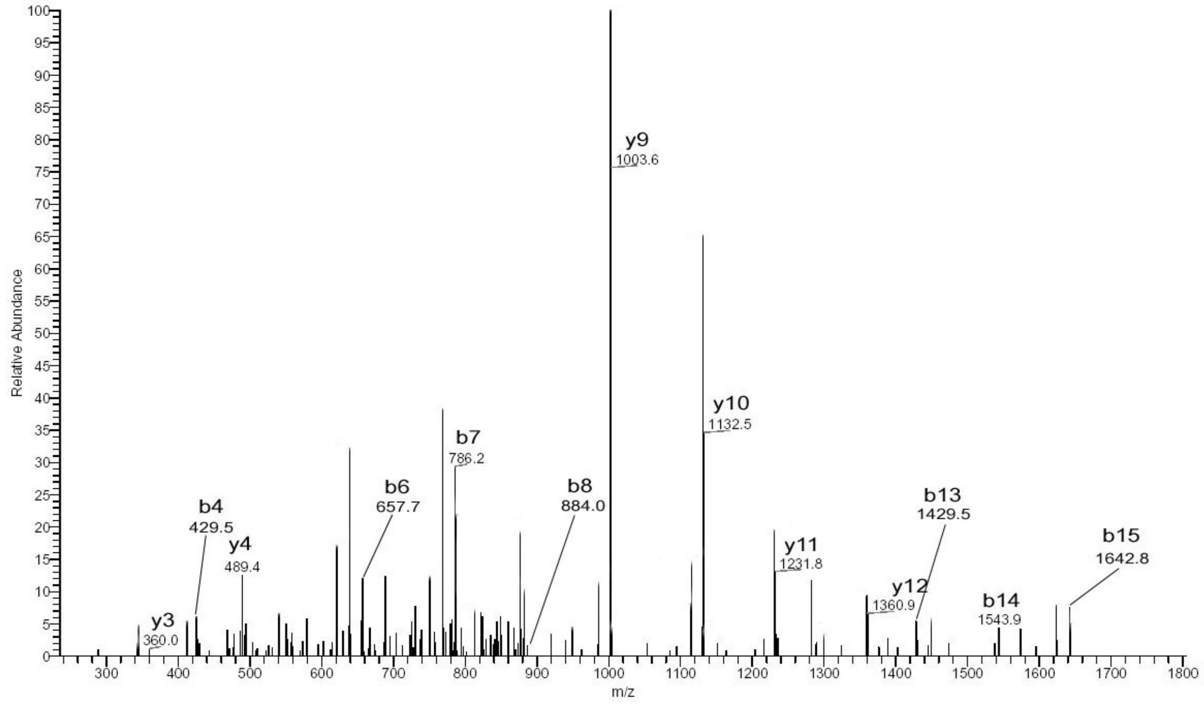

075915 **PRA1 family protein 3** MH+ (Da): 1312.8985

b ions			y ions		
b1	A	72.09	y11	AWDDFFPGSDR	1313.36
b2	AW	**258.31**	y10	WDDFFPGSDR	1242.28
b3	AWD	**373.4**	y9	DDFFPGSDR	**1056.07**
b4	AWDD	488.48	y8	DFFPGSDR	**940.98**
b5	AWDDF	**635.66**	y7	FFPGSDR	**825.89**
b6	AWDDFF	**782.84**	y6	FPGSDR	**678.71**
b7	AWDDFFP	**879.95**	y5	PGSDR	**531.54**
b8	AWDDFFPG	937.01	y4	GSDR	**434.42**
b9	AWDDFFPGS	**1024.08**	y3	SDR	377.37
b10	AWDDFFPGSD	1139.17	y2	DR	290.29
b11	AWDDFFPGSDR	1295.36	y1	R	175.2

The detected ions were shown in bold.

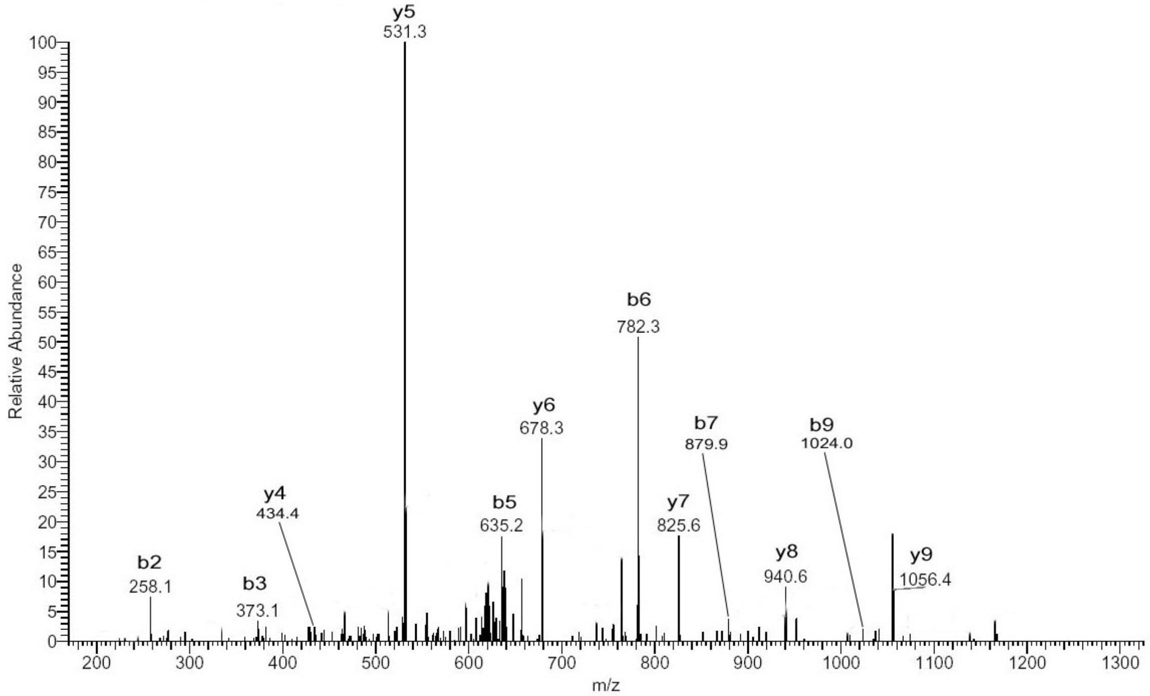

P60709 **Actin, cytoplasmic 1** MH+ (Da): 1132.7391

b ions y ions

b1	G	58.07			
b2	GY	**221.24**	y10	GYSFTTTAER	1133.2
b3	GYS	**308.32**	y9	YSFTTTAER	1076.14
b4	GYSF	**455.5**	y8	SFTTTAER	**912.97**
b5	GYSFT	**556.6**	y7	FTTTAER	**825.89**
b6	GYSFTT	**657.71**	y6	TTTAER	**678.71**
b7	GYSFTTT	**758.81**	y5	TTAER	**577.61**
b8	GYSFTTTA	**829.89**	y4	TAER	**476.5**
b9	GYSFTTTAE	959.01	y3	AER	**375.4**
b10	GYSFTTTAER	1115.2	y2	ER	**304.32**
			y1	R	**175.2**

The detected ions were shown in bold.

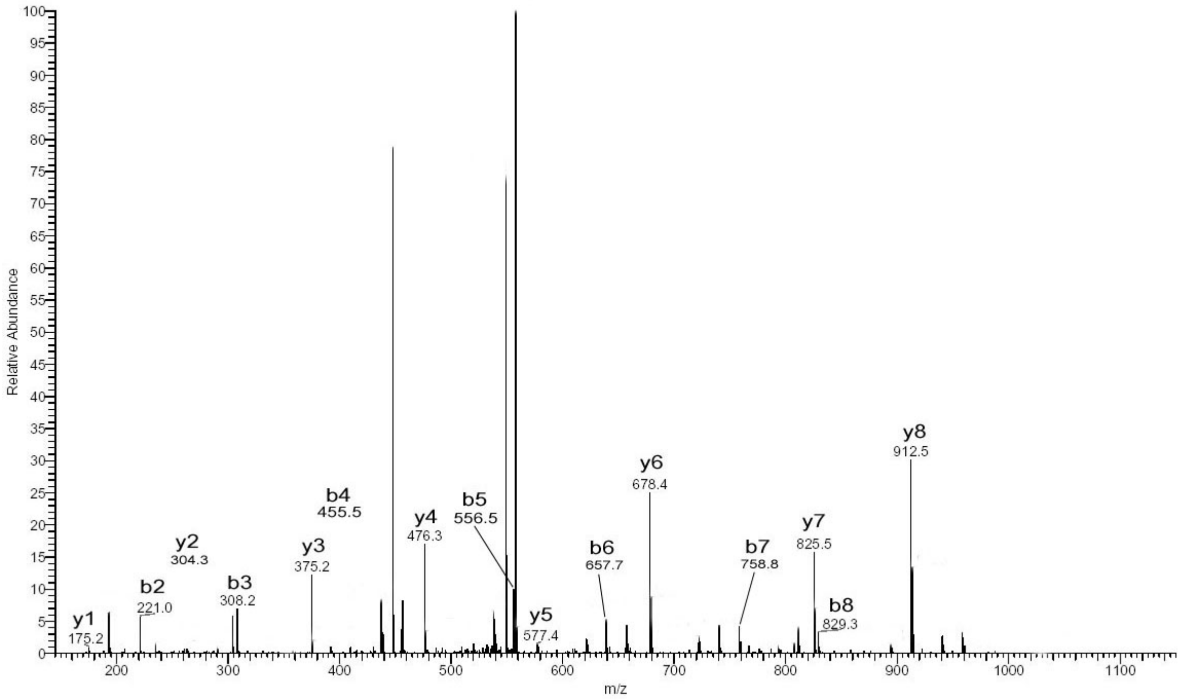

A8MWI1	**Probable G-protein coupled receptor 179 precursor**		MH+ (Da):	1150.9204	
b ions			y ions		
b1	G	58.07	y12	GSSEAAGSVETR	1151.17
b2	GS	145.15	y11	SSEAAGSVETR	1094.12
b3	GSS	232.22	y10	SEAAGSVETR	**1007.04**
b4	GSSE	**361.34**	y9	EAAGSVETR	919.96
b5	GSSEA	**432.42**	y8	AAGSVETR	790.84
b6	GSSEAA	**503.5**	y7	AGSVETR	**719.76**
b7	GSSEAAG	**560.55**	y6	GSVETR	**648.69**
b8	GSSEAAGS	**647.63**	y5	SVETR	591.63
b9	GSSEAAGSV	746.76	y4	VETR	**504.56**
b10	GSSEAAGSVE	**875.87**	y3	ETR	405.42
b11	GSSEAAGSVET	976.98	y2	TR	**276.31**
b12	GSSEAAGSVETR	1133.17	y1	R	175.2

The detected ions were shown in bold.

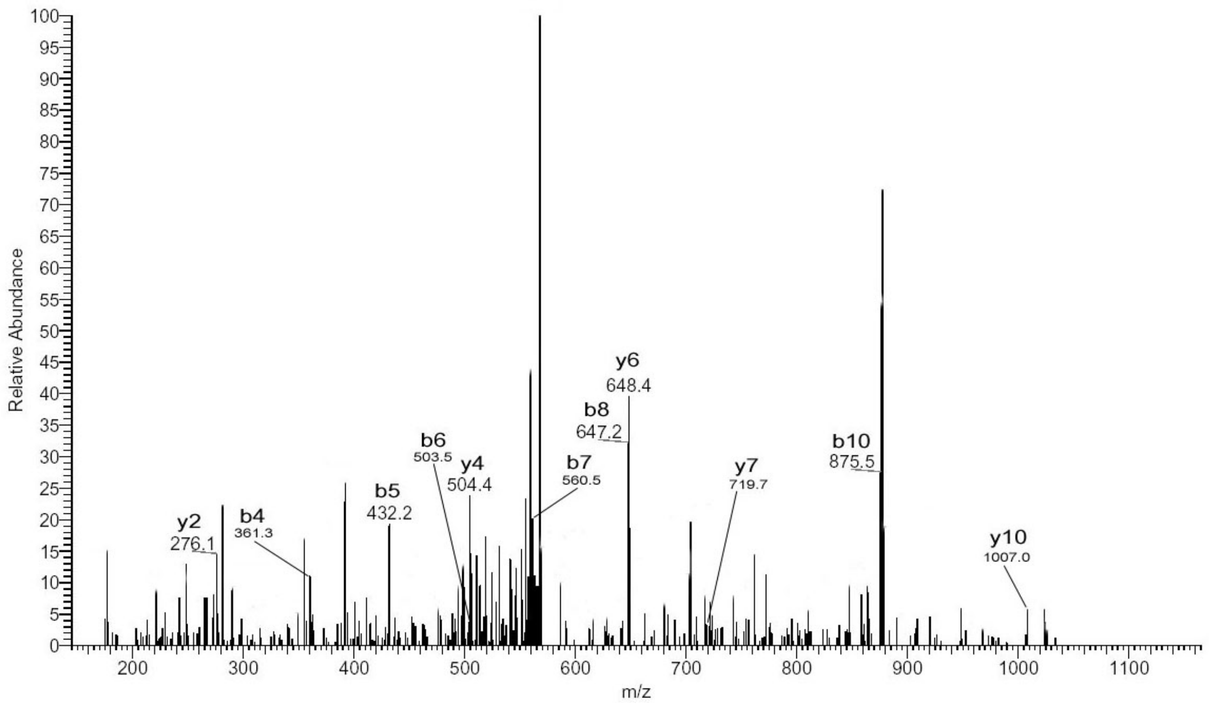

P38646	**heat shock 70kDa protein 9, mitochondrial precursor**		MH+ (Da):	1450.7788	
b ions			y ions		
b1	T	102.12	y14	TTPSVVAFTADGER	1451.57
b2	TT	203.23	y13	TPSVVAFTADGER	1350.46
b3	TTP	300.34	y12	PSVVAFTADGER	1249.36
b4	TTPS	387.42	y11	SVVAFTADGER	1152.24
b5	TTPSV	486.55	y10	VVAFTADGER	1065.16
b6	TTPSVV	585.69	y9	VAFTADGER	**966.03**
b7	TTPSVVA	656.76	y8	AFTADGER	**866.9**
b8	TTPSVVAF	803.94	y7	FTADGER	**795.82**
b9	TTPSVVAFT	905.05	y6	TADGER	**648.64**
b10	TTPSVVAFTA	976.12	y5	ADGER	**547.54**
b11	TTPSVVAFTAD	1091.21	y4	DGER	**476.46**
b12	TTPSVVAFTADG	1148.27	y3	GER	**361.37**
b13	TTPSVVAFTADGE	1277.38	y2	ER	304.32
b14	TTPSVVAFTADGER	1433.57	y1	R	175.2

The detected ions were shown in bold.

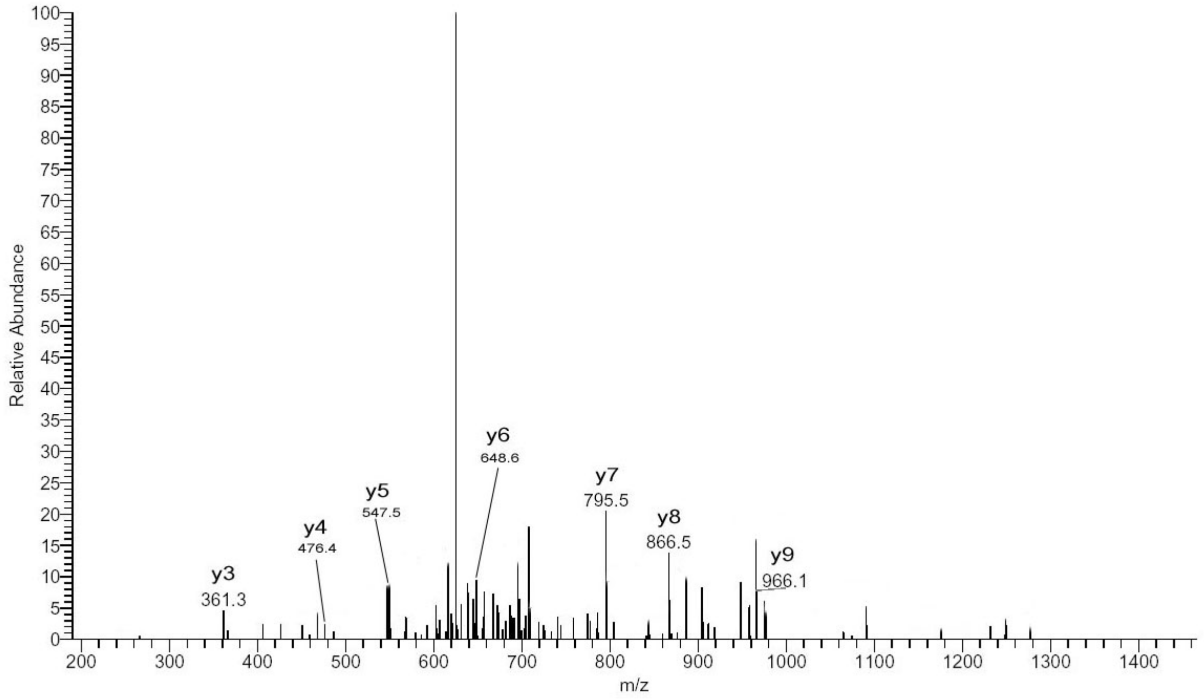

Q9BSJ8	**Isoform 1 of Protein FAM62A**		MH+ (Da):	1402.3521	
b ions			y ions		
b1	Q	129.15	y12	QLLDDEEQLTAK	1403.52
b2	QL	242.31	y11	LLDDEEQLTAK	1275.39
b3	QLL	**355.46**	y10	LDDEEQLTAK	**1162.23**
b4	QLLD	**470.55**	y9	DDEEQLTAK	**1049.07**
b5	QLLDD	585.64	y8	DEEQLTAK	**933.98**
b6	QLLDDE	714.76	y7	EEQLTAK	**818.89**
b7	QLLDDEE	843.87	y6	EQLTAK	689.78
b8	QLLDDEEQ	972	y5	QLTAK	**560.66**
b9	QLLDDEEQL	1085.16	y4	LTAK	**432.53**
b10	QLLDDEEQLT	1186.27	y3	TAK	**319.37**
b11	QLLDDEEQLTA	**1257.35**	y2	AK	218.27
b12	QLLDDEEQLTAK	1385.52	y1	K	147.19

The detected ions were shown in bold.

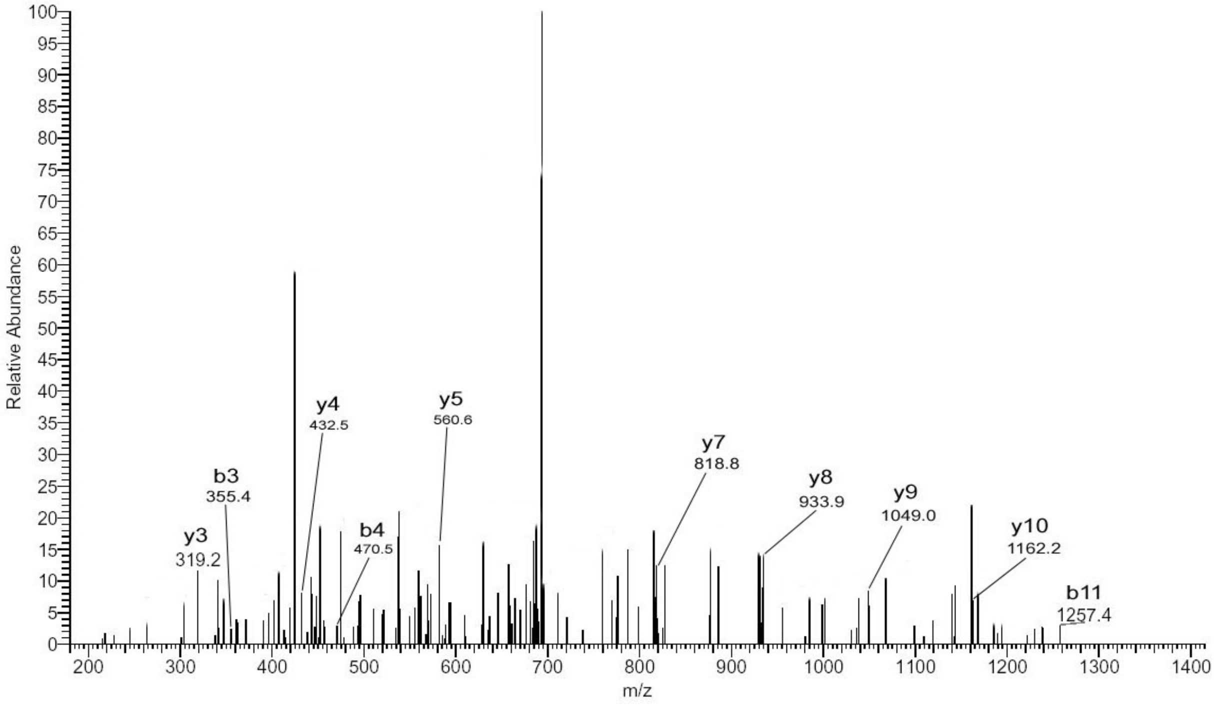

Q7Z5G9	**ELMO2 protein**		MH+ (Da)：	1484.9090	
b ions			y ions		
b1	M	132.21	y12	MERTQSSNMETR	1470.61
b2	ME	261.32	y11	ERTQSSNMETR	1339.41
b3	MER	417.51	y10	RTQSSNMETR	**1210.3**
b4	MERT	**518.62**	y9	TQSSNMETR	**1054.11**
b5	MERTQ	646.75	y8	QSSNMETR	953.01
b6	MERTQS	**733.82**	y7	SSNMETR	824.88
b7	MERTQSS	820.9	y6	SNMETR	**737.8**
b8	MERTQSSN	**935.01**	y5	NMETR	**650.72**
b9	MERTQSSNM	**1066.2**	y4	METR	**536.62**
b10	MERTQSSNME	**1195.31**	y3	ETR	**405.42**
b11	MERTQSSNMET	1296.42	y2	TR	**276.31**
b12	MERTQSSNMETR	1452.61	y1	R	175.2

The detected ions were shown in bold.

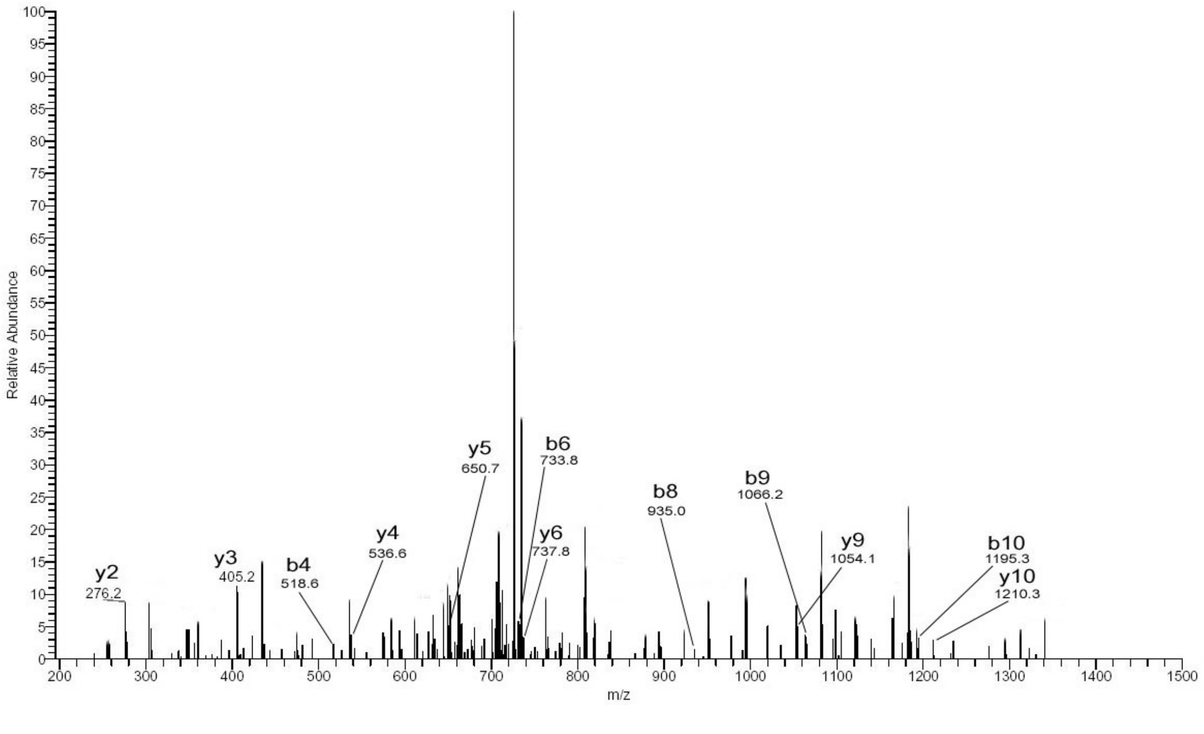

	Isoform 1 of Coiled-coil domain-containing protein 73		MH+ (Da):	1583.8480	
Q6ZRK6 b ions			y ions		
b1	E	130.13	y13	EAMAVFKKQLQMK	1568.93
b2	EA	201.21	y12	AMAVFKKQLQMK	1439.81
b3	EAM	348.4	y11	MAVFKKQLQMK	1368.73
b4	EAMA	419.48	y10	AVFKKQLQMK	1237.54
b5	EAMAV	518.61	y9	VFKKQLQMK	1166.46
b6	EAMAVF	**665.79**	y8	FKKQLQMK	1067.33
b7	EAMAVFK	793.96	y7	KKQLQMK	**920.15**
b8	EAMAVFKK	**922.14**	y6	KQLQMK	791.98
b9	EAMAVFKKQ	**1050.27**	y5	QLQMK	663.8
b10	EAMAVFKKQL	1163.43	y4	LQMK	**535.67**
b11	EAMAVFKKQLQ	1291.56	y3	QMK	422.51
b12	EAMAVFKKQLQM	1422.75	y2	MK	294.38
b13	EAMAVFKKQLQMK	1550.93	y1	K	147.19

The detected ions were shown in bold.

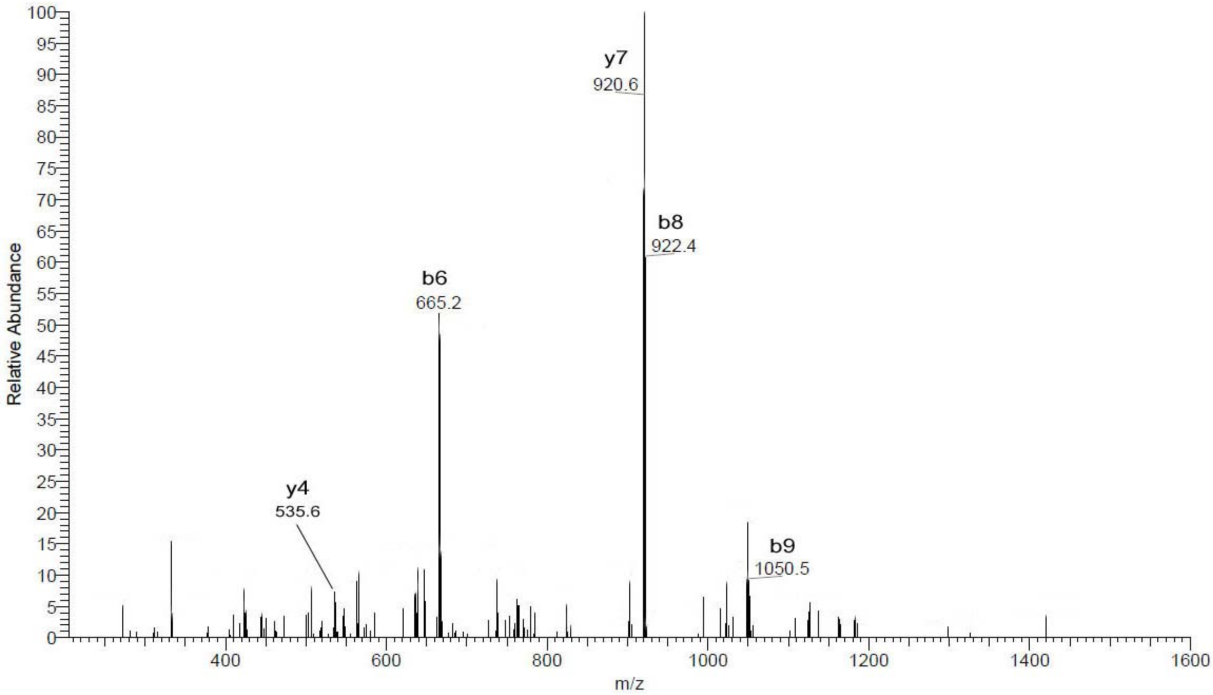

P41219	**Isoform 1 of Peripherin**		MH+ (Da)：	1309.9330	
b ions			y ions		
b1	L	114.17	y8	LLEGEESR	933
b2	LL	**227.33**	y7	LEGEESR	**819.84**
b3	LLE	**356.45**	y6	EGEESR	**706.68**
b4	LLEG	413.5	y5	GEESR	**577.56**
b5	LLEGE	542.62	y4	EESR	**520.51**
b6	LLEGEE	**671.73**	y3	ESR	**391.4**
b7	LLEGEES	758.81	y2	SR	**262.28**
b8	LLEGEESR	915	y1	R	175.2

The detected ions were shown in bold.

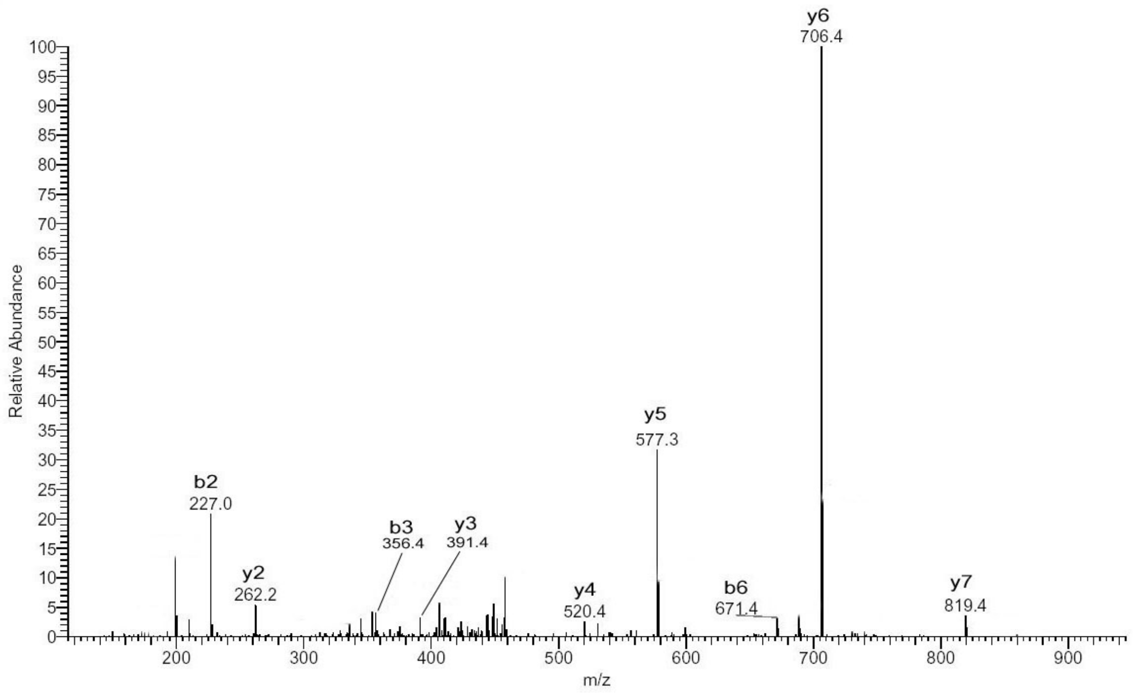

P25705	**ATP synthase subunit alpha, mitochondrial precursor**		MH+ (Da):	1316.9060	
b ions			y ions		
b1	T	102.12	y12	TSIAIDTIINQK	1317.52
b2	TS	189.2	y11	SIAIDTIINQK	1216.41
b3	TSI	302.36	y10	IAIDTIINQK	1129.33
b4	TSIA	**373.44**	y9	AIDTIINQK	**1016.17**
b5	TSIAI	**486.6**	y8	IDTIINQK	**945.1**
b6	TSIAID	**601.68**	y7	DTIINQK	**831.94**
b7	TSIAIDT	702.79	y6	TIINQK	**716.85**
b8	TSIAIDTI	**815.95**	y5	IINQK	**615.74**
b9	TSIAIDTII	**929.11**	y4	INQK	**502.58**
b10	TSIAIDTIIN	**1043.21**	y3	NQK	**389.42**
b11	TSIAIDTIINQ	1171.34	y2	QK	**275.32**
b12	TSIAIDTIINQK	1299.52	y1	K	147.19

The detected ions were shown in bold.

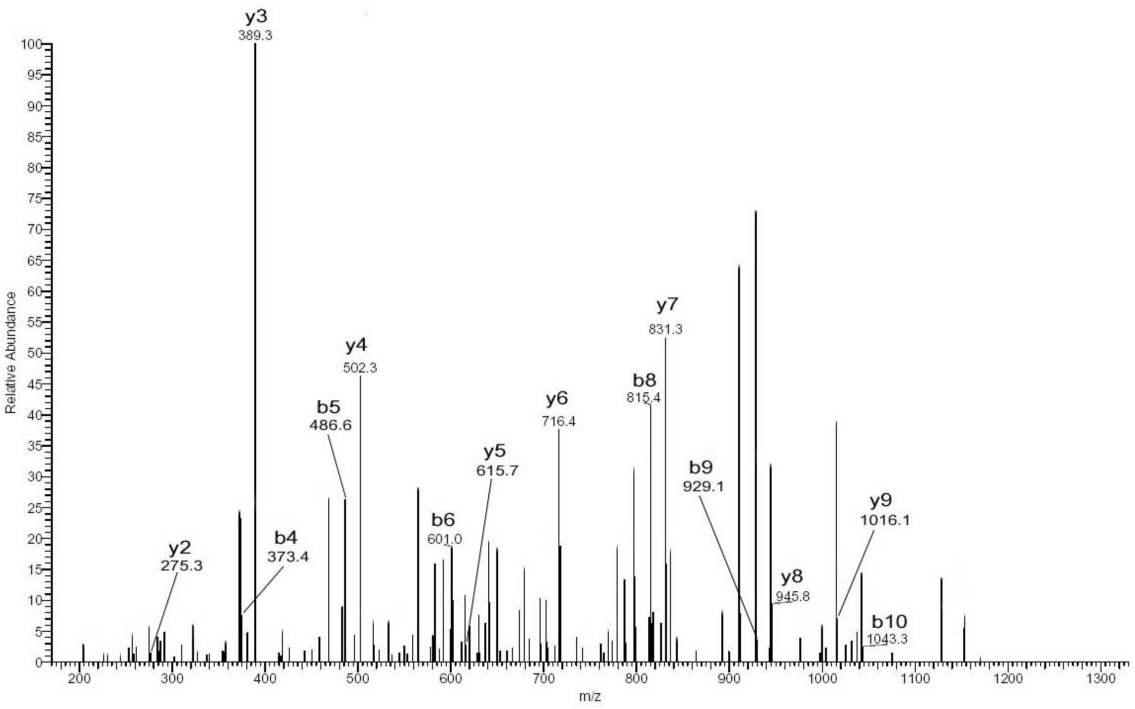

B4DGP8	**Calnexin precursor**		MH+ (Da):	1488.7949	
b ions			y ions		
b1	I	114.17	y13	IVDDWANDGWGLK	1489.62
b2	IV	213.31	y12	VDDWANDGWGLK	1376.46
b3	IVD	**328.4**	y11	DDWANDGWGLK	**1277.33**
b4	IVDD	**443.48**	y10	DWANDGWGLK	1162.24
b5	IVDDW	**629.7**	y9	WANDGWGLK	1047.15
b6	IVDDWA	**700.78**	y8	ANDGWGLK	**860.94**
b7	IVDDWAN	814.88	y7	NDGWGLK	**789.86**
b8	IVDDWAND	**929.97**	y6	DGWGLK	**675.75**
b9	IVDDWANDG	987.02	y5	GWGLK	**560.67**
b10	IVDDWANDGW	1173.23	y4	WGLK	**503.61**
b11	IVDDWANDGWG	**1230.29**	y3	GLK	317.4
b12	IVDDWANDGWGL	1343.45	y2	LK	260.35
b13	IVDDWANDGWGLK	1471.62	y1	K	147.19

The detected ions were shown in bold.

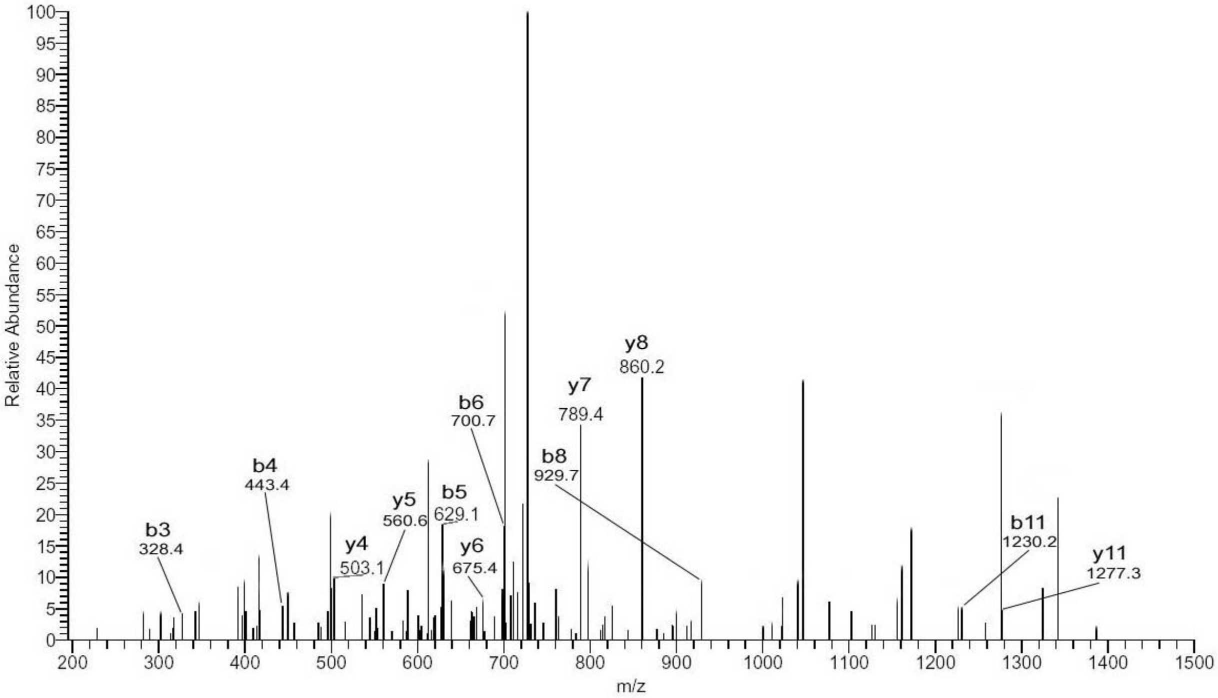

Q7Z591	**Isoform 1 of AT-hook-containing transcription factor**		MH+ (Da)：	1236.9160	
b ions			y ions		
b1	P	98. 13	y12	PLPRQGATLAGR	1237. 44
b2	PL	211. 29	y11	LPRQGATLAGR	1140. 32
b3	PLP	308. 41	y10	PRQGATLAGR	1027. 16
b4	PLPR	464. 6	y9	RQGATLAGR	930. 05
b5	PLPRQ	**592. 73**	y8	QGATLAGR	773. 86
b6	PLPRQG	649. 78	y7	GATLAGR	**645. 73**
b7	PLPRQGA	**720. 86**	y6	ATLAGR	588. 68
b8	PLPRQGAT	821. 96	y5	TLAGR	**517. 6**
b9	PLPRQGATL	**935. 12**	y4	LAGR	416. 49
b10	PLPRQGATLA	**1006. 2**	y3	AGR	**303. 33**
b11	PLPRQGATLAG	**1063. 25**	y2	GR	**232. 25**
b12	PLPRQGATLAGR	1219. 44	y1	R	175. 2

The detected ions were shown in bold.

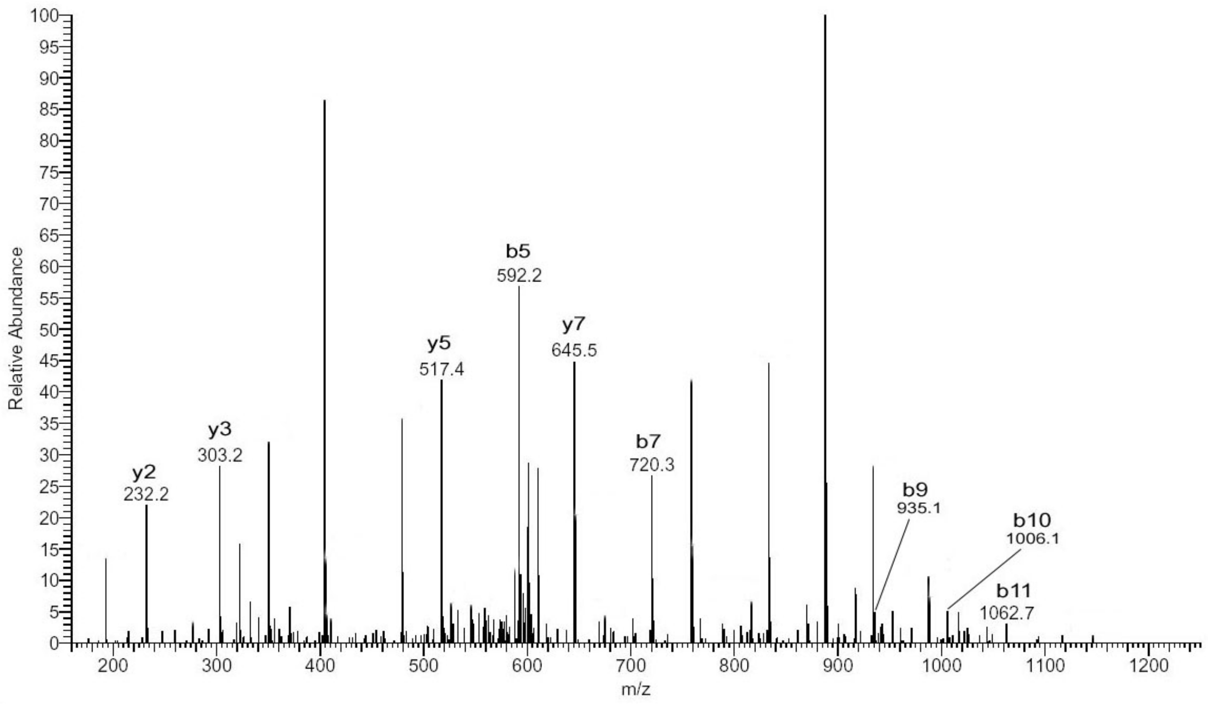

P05107	**Integrin beta-2 precursor**		MH+ (Da):	1090.0657	
b ions			y ions		
b1	A	72.09	y10	ALNEITESGR	1090.17
b2	AL	**185.25**	y9	LNEITESGR	1019.09
b3	ALN	**299.36**	y8	NEITESGR	**905.93**
b4	ALNE	**428.47**	y7	EITESGR	**791.83**
b5	ALNEI	541.63	y6	ITESGR	**662.71**
b6	ALNEIT	**642.74**	y5	TESGR	549.55
b7	ALNEITE	771.85	y4	ESGR	**448.45**
b8	ALNEITES	**858.93**	y3	SGR	**319.33**
b9	ALNEITESG	915.98	y2	GR	**232.25**
b10	ALNEITESGR	1072.17	y1	R	175.2

The detected ions were shown in bold.

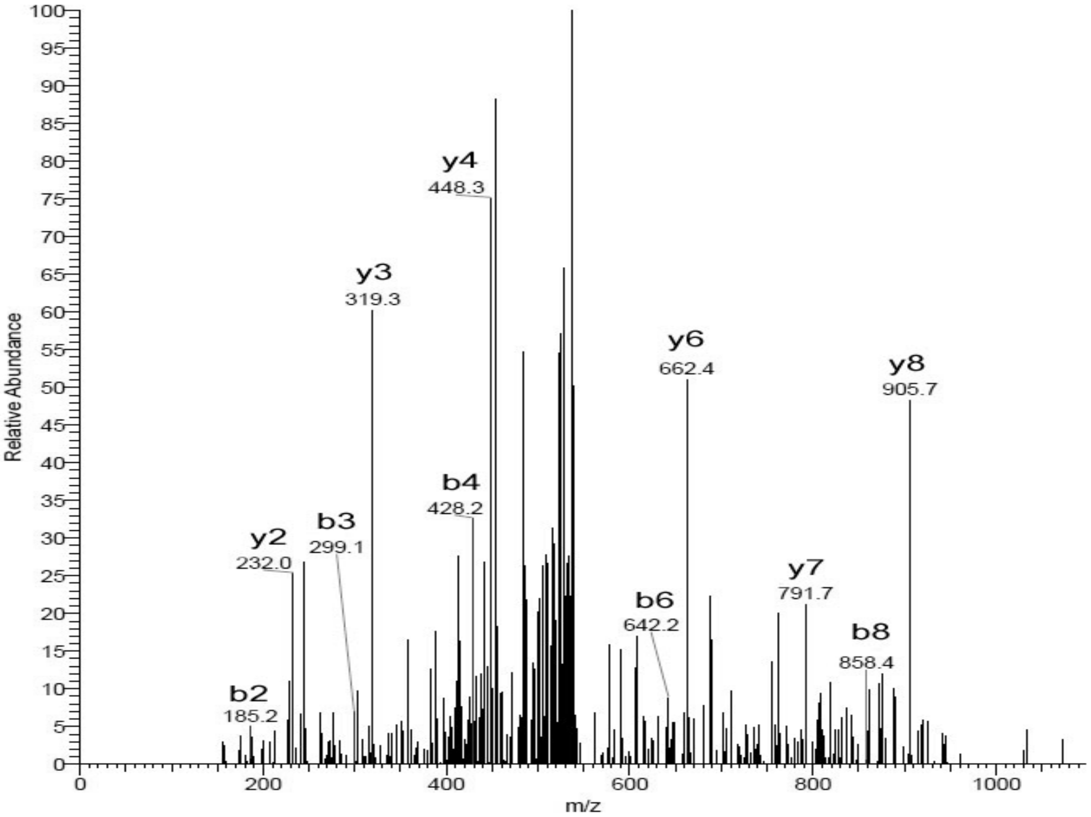

Figure S3

The MS/MS spectra for single-peptide-based identifications. The detected b and y ions used in the protein identification were labeled in the MS/MS spectra. The unlabeled peaks are a, c, x or z ions generated in mass spectrometer.
